# von Willebrand factor links primary hemostasis to innate immunity

**DOI:** 10.1038/s41467-022-33796-7

**Published:** 2022-11-03

**Authors:** Clive Drakeford, Sonia Aguila, Fiona Roche, Karsten Hokamp, Judicael Fazavana, Mariana P. Cervantes, Annie M. Curtis, Heike C. Hawerkamp, Sukhraj Pal Singh Dhami, Hugo Charles-Messance, Emer E. Hackett, Alain Chion, Soracha Ward, Azaz Ahmad, Ingmar Schoen, Eamon Breen, Joe Keane, Ross Murphy, Roger J. S. Preston, Jamie M. O’Sullivan, Frederick J. Sheedy, Padraic Fallon, James S. O’Donnell

**Affiliations:** 1 Irish Centre for Vascular Biology, School of Pharmacy and Biomolecular Science (PBS), Royal College of Surgeons in Ireland, Dublin, Ireland; 2 Centro Regional de Hemodonación, Hospital Universitario Morales Meseguer, IMIB-Arrixaca, Murcia, Spain; 3 Smurfit Institute of Genetics, School of Genetics and Microbiology, Trinity College Dublin, College Green, Dublin, Ireland; 4 School of Pharmacy and Biomolecular Science (PBS) and Tissue Engineering Research Group (TERG), Royal College of Surgeons in Ireland, Dublin, 2 Ireland; 5 School of Medicine, Trinity College Dublin, Dublin, 2 Ireland; 6 School of Biochemistry and Immunology, Trinity Biomedical Sciences Institute, Trinity College Dublin, Dublin, Ireland; 7 Department of Clinical Medicine, Trinity Translational Medicine Institute, Trinity College Dublin, Dublin, Ireland; 8 Department of Cardiology, St James’s Hospital, Dublin, Ireland; 9 National Children’s Research Centre, Our Lady’s Children’s Hospital, Dublin, Ireland; 10 National Coagulation Centre, St James’s Hospital, Dublin, Ireland

**Keywords:** Thrombosis, Molecular medicine, Monocytes and macrophages, Innate immunity

## Abstract

The plasma multimeric glycoprotein von Willebrand factor (VWF) plays a critical role in primary hemostasis by tethering platelets to exposed collagen at sites of vascular injury. Recent studies have identified additional biological roles for VWF, and in particular suggest that VWF may play an important role in regulating inflammatory responses. However, the molecular mechanisms through which VWF exerts its immuno-modulatory effects remain poorly understood. In this study, we report that VWF binding to macrophages triggers downstream MAP kinase signaling, NF-κB activation and production of pro-inflammatory cytokines and chemokines. In addition, VWF binding also drives macrophage M1 polarization and shifts macrophage metabolism towards glycolysis in a p38-dependent manner. Cumulatively, our findings define an important biological role for VWF in modulating macrophage function, and thereby establish a novel link between primary hemostasis and innate immunity.

## Abstract (web summary)

von Willebrand factor (VWF) plays a critical role in primary hemostasis following vascular injury by tethering platelets to exposed collagen. Here, VWF binding to macrophages is shown to trigger NF-κB activation and induce pro-inflammatory responses.

## Introduction

von Willebrand factor (VWF) is a large sialoglycoprotein that circulates in normal plasma as a series of heterogeneous multimers^1,2^. For many years, the importance of plasma VWF in maintaining normal hemostasis has been recognized^3,4^. VWF binds to exposed subendothelial collagen at sites of vascular injury^5^. Subsequently, shear stress-induced unwinding of globular VWF results in exposure of the platelet glycoprotein Ibα (GPIbα) binding site within the A1 domain^5,6^. Consequently, tethered and unwound VWF can recruit platelets to the site of injury, leading to the formation of the primary platelet plug. In addition, VWF also binds with high affinity to procoagulant factor VIII, thereby protecting it against proteolysis and premature clearance^7^.

Besides its hemostatic function, recent studies have identified additional biological roles for VWF, including inhibition of angiogenesis and promotion of tumor cell apoptosis^8,9^. Furthermore, accumulating evidence suggests that VWF plays important roles in enhancing inflammatory responses^10–17^. Acute activation of endothelial cells (EC) triggers the secretion of high molecular weight multimeric (HMWM) VWF stored within Weibel Palade bodies (WPB)^1^. Consequently, it is perhaps unsurprising that elevated plasma VWF levels have been reported in association with different types of sepsis, as well as a number of other vascular pathologies^10^. Indeed plasma VWF:Ag and VWF propeptide (VWFpp) levels have both been proposed as useful biomarkers that correlate with severity and/or clinical outcomes in a number of different disease settings, including COVID-19, cerebral malaria, sickle cell disease, systemic inflammatory response syndrome and a variety of different cancers^9,18–21^.

Importantly, data from studies conducted in a number of different animal inflammatory disease models suggest that VWF does not merely serve as a marker of acute EC activation, but rather that it plays an active role in mediating the underlying pathophysiology^12–15,18,22,23^. For example, in a caecal puncture sepsis model, overall survival was significantly increased in VWF-deficient mice compared to wild-type controls^14^. In addition, Petri et al showed that VWF-blocking antibodies significantly attenuated neutrophil recruitment into thioglycollate-inflamed peritoneum and keratinocyte-derived chemokine (KC)-stimulated exposed cremaster muscle^13^. In both of these murine models of inflammation, VWF-modulated neutrophil extravasation was dependent upon the presence of platelets. Similarly, VWF-blocking antibodies were shown to again significantly reduce neutrophil recruitment in murine models of immune-complex-mediated vasculitis and irritative contact dermatitis respectively^12^. Interestingly, VWF-modulated neutrophil recruitment in these murine models of cutaneous inflammation was mediated through a platelet-independent pathway^12^.

All together, these findings demonstrate that VWF influences multiple different aspects of inflammation. However, the molecular mechanisms through which VWF exerts its pro-inflammatory effects remain poorly understood. Recent studies have reported that VWF can bind to macrophages, following which it is rapidly endocytosed^24,25^. Moreover, hepatic Kupffer cells have been shown to play a role in regulating the circulatory half-life of plasma VWF^24,26^. A number of specific macrophage receptors have also been implicated in regulating VWF binding, including the low-density lipoprotein receptor-related protein-1 (LRP1), the scavenger receptor class A member 1 (SR-A1), macrophage galactose-type lectin (MGL) and Siglec-5^26–29^. In this paper, we investigate the hypothesis that the binding of multimeric VWF to macrophages may influence innate inflammatory responses. We demonstrate that VWF binding triggers significant intracellular signaling within macrophages, resulting in p-38 and subsequent HIF-1α activation, together with enhanced glycolysis. This VWF interaction is mediated in part through macrophage LRP1, and leads to the secretion of proinflammatory cytokines and chemokines. Collectively, these data define a biological role for VWF in linking primary hemostasis to innate immunity at sites of vascular injury.

## Results

### VWF binding to macrophages triggers pro-inflammatory signaling

**Fig. 1:**
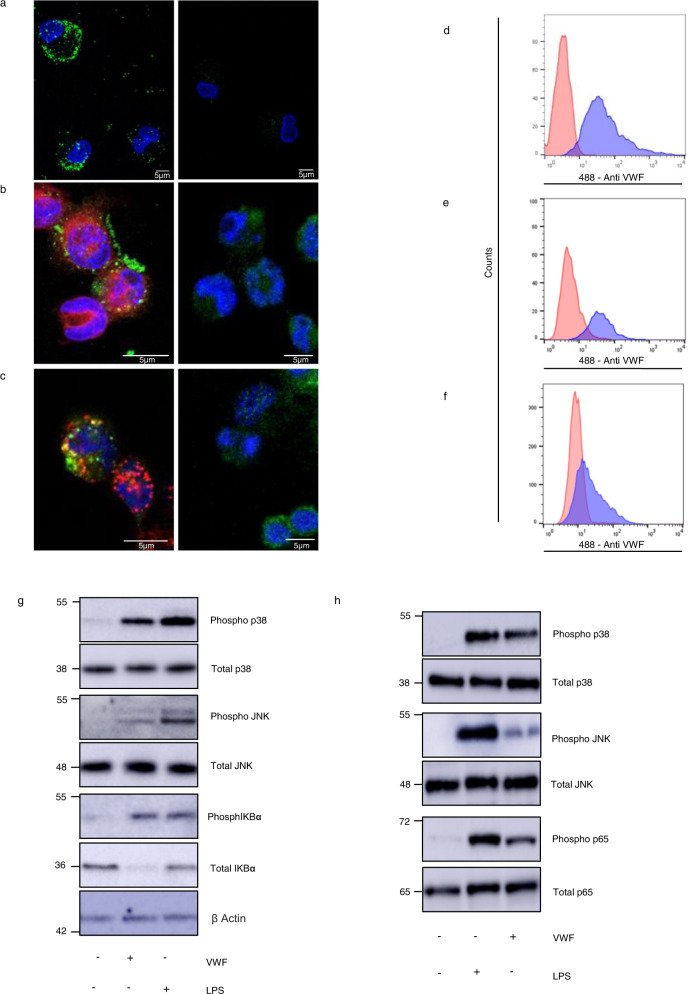
VWF binds to macrophages and triggers inflammatory signaling. **a** Binding of plasma-derived VWF (pd-VWF) to primary human macrophages and **b** THP1 macrophages was assessed in vitro using confocal microscopy as detailed in Materials and Methods (VWF staining in green; nuclear DAPI staining in blue; cell membrane staining in red). **c** VWF internalization was assessed using THP1 macrophage and anti-VWF antibody staining in green, DAPI and anti-EEA1 (early endosomes antigen 1) antibody staining in red and co-localization in yellow. pd-VWF or recombinant VWF (300–600 nM) were incubated with macrophages for 30 min at 37 °C and cells analyzed by flow cytometry. Representative histograms are presented where red represents control cells not treated with VWF and blue cells treated with VWF. **d** pd-VWF and **e** recombinant VWF binding to primary human macrophages; **f** recombinant VWF binding to THP1 macrophages. Flow gating strategy is presented in Supplementary Fig. 14.Western blot analysis of phosphorylation of p38, JNK, IKBα, and p65 in **g** primary human and **h** primary murine macrophages incubated with VWF (10 µg/ml) or LPS (100 ng/ml) for 30 min. All scale bars are 5 μM. All experiments were performed in triplicate and source data for this figure are provided as a Source Data file.

Recent studies have reported shear-dependent binding of VWF to macrophages^30,31^. In preliminary studies, we observed that purified plasma-derived (pd)-VWF can also bind to primary human macrophages and THP-1-derived macrophages under static conditions (Fig. 1a, b). Moreover, this static binding was followed by VWF endocytosis (Fig. 1c). Flow cytometry studies confirmed that pd-VWF and recombinant VWF both bound to human macrophages (Fig. 1d, f). In contrast, no VWF binding was observed to primary human monocytes or THP1 cells that had not been pre-activated with PMA (Supplementary Fig. 1A)^31^. Importantly, we further observed that pd-VWF binding was associated with pro-inflammatory intracellular signaling in both primary human macrophages (Fig. 1g) and in murine bone marrow-derived macrophages (BMDMs) (Fig. 1h). Both VWF binding to macrophages and pro-inflammatory effects were dose-dependent in nature (Supplementary Fig. 2). In particular, VWF induced activation of the MAPKinase pro-inflammatory signaling pathway with phosphorylation of p38 and JNK. In addition, VWF binding also activated NF-κB with phosphorylation of its regulatory subunit IKBα (Fig. 1g, h and Supplementary Fig. 1B).

**Fig. 2:**
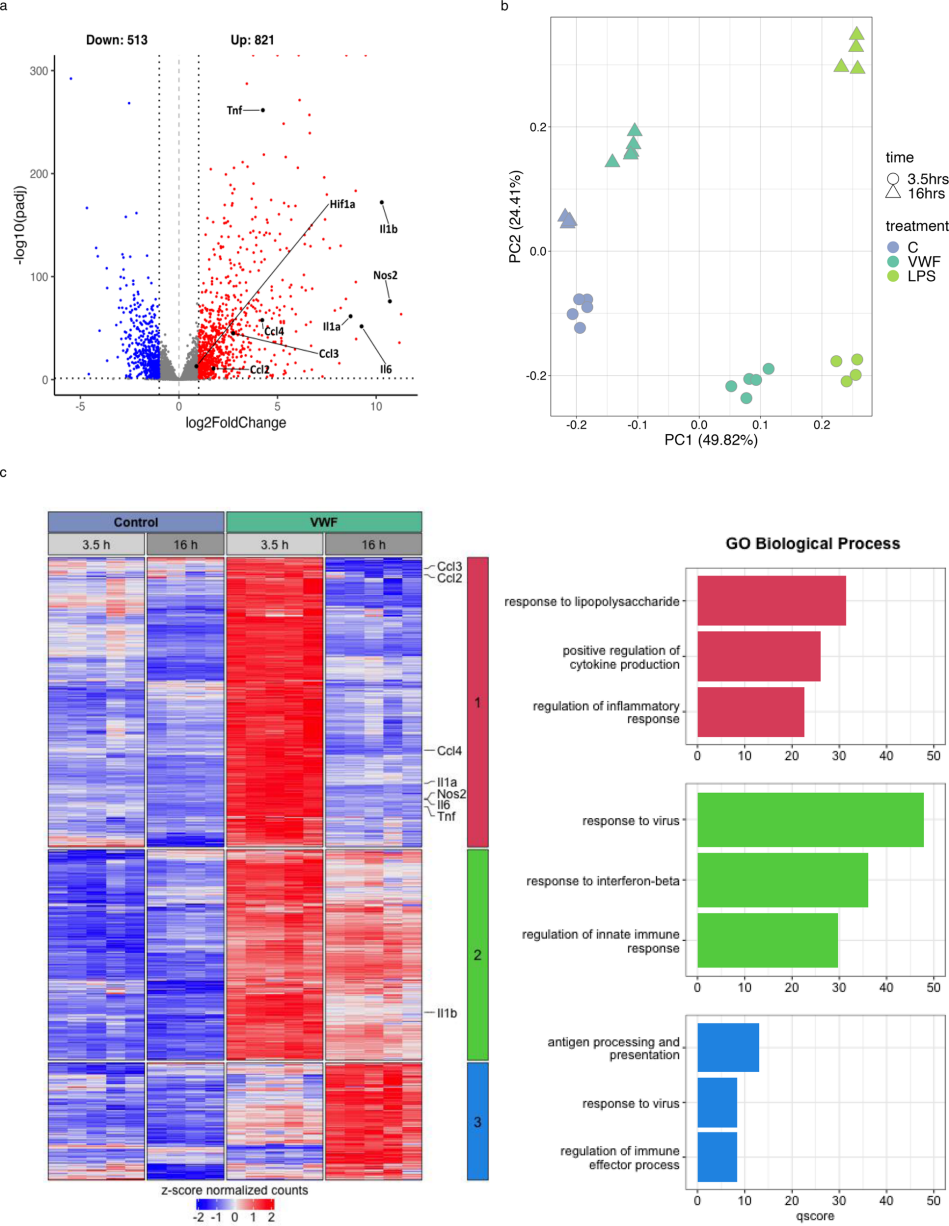
VWF induces a pro-inflammatory transcriptional response in macrophages. **a** Volcano plot of significantly differentially expressed genes (FDR < 0.05) as detected by RNAseq in VWF-treated macrophages versus PBS-control for 3.5 h (*n* = 5 each). Red and blue dots indicate statistically significant up- and down-regulated genes, respectively. Key pro-inflammatory genes are labeled in black. **b** First (PC1) and second (PC2) principle components of a PCA plot of RNA-seq data of control- (blue), VWF-(dark green) and LPS-treated (light green) macrophages at different time points. **c** Heatmap of genes that are induced by VWF-treated macrophages versus control. Genes were clustered using hierarchical clustering with Pearson’s correlation similarity and complete linkage using z-score transformed gene expression values. Key proinflammatory genes are listed along the heatmap. The top three clusters were identified using k-means clustering (center, colored bar). Corresponding gene ontology enrichment results from these clusters are provided as bar charts on the right. The q-score represents the negative log_10_ of the *p*-adjusted value. Source data for this figure are provided as a Source Data file.

To further investigate VWF signaling effects, RNAseq studies were performed on BMDMs after incubation with pd-VWF (10 μg/ml), LPS (100 ng/ml) or PBS control for 3.5 h (T1) or 16 h (T2) respectively. At 3.5 h, we observed that 1334 genes were differentially expressed (*p*-adjusted value < 0.05, absolute fold change > 2; 821 upregulated, 513 downregulated) in macrophages treated with VWF compared to untreated control cells (Fig. 2a). Pertinently, pro-inflammatory cytokine (TNF, IL-6 and IL-1β) and chemokine (CCL2, CCL3, and CCL4) expression were all significantly increased following VWF exposure. The RNAseq data were confirmed using qPCR for selected pro-inflammatory genes (Supplementary Fig. 3A–F). Together, the transcriptomic analysis confirms that VWF treatment of macrophages is associated with a significant early pro-inflammatory effect. Importantly, however, this response differed both qualitatively and quantitatively compared to that observed following LPS treatment for the same time period (VWF versus LPS for 3.5 h −1005 differentially expressed genes; 441 upregulated, 564 downregulated; Supplementary Fig. 4A).

While both VWF and LPS had pro-inflammatory effects on macrophages at 3.5 h, principal component analysis (PCA) highlighted marked differences in transcriptomic reprogramming induced by either VWF or LPS treatment at 16 h (Fig. 2b). At this later time point, LPS treatment of macrophages was still associated with pro-inflammatory gene expression (Supplementary Fig. 4B), whereas macrophages treated with VWF reverted to an expression profile similar to that of the control group (Fig. 2b). Analysis of the genes induced by VWF across both time points highlights three main waves of transcriptional expression: (Cluster 1) transient early response genes that show expression at 3.5 h but are switched off at 16 h, (Cluster 2) early response genes that show sustained expression at the later time point, albeit at a reduced level, and (Cluster 3) late response genes that are switched on at 16 h. Gene ontology enrichment analysis of these induced gene clusters revealed a strong enrichment for genes involved in proinflammatory processes (Fig. 2c). Many key pro-inflammatory genes (including TNF, IL-6, IL-1β, CCL2, CCL3, and CCL4) lie within clusters 1 and 2 and show attenuated expression following VWF treatment for 16 h compared to VWF treatment for only 3.5 h (Fig. 2c and Supplementary Fig. 5A, B). Overall, we observed that 2249 genes (998 upregulated, 1251 downregulated) were differentially expressed following macrophage treatment with VWF versus LPS for 16 h (Supplementary Fig. 4C, D). Collectively, these findings demonstrate that the pro-inflammatory effects of VWF on macrophages are qualitatively distinct from those induced by LPS and further highlight that VWF-induced macrophage transcriptomic reprogramming is subject to temporal regulation.

### VWF induces cytokine and chemokine expression and promotes monocyte chemotaxis

**Fig. 3:**
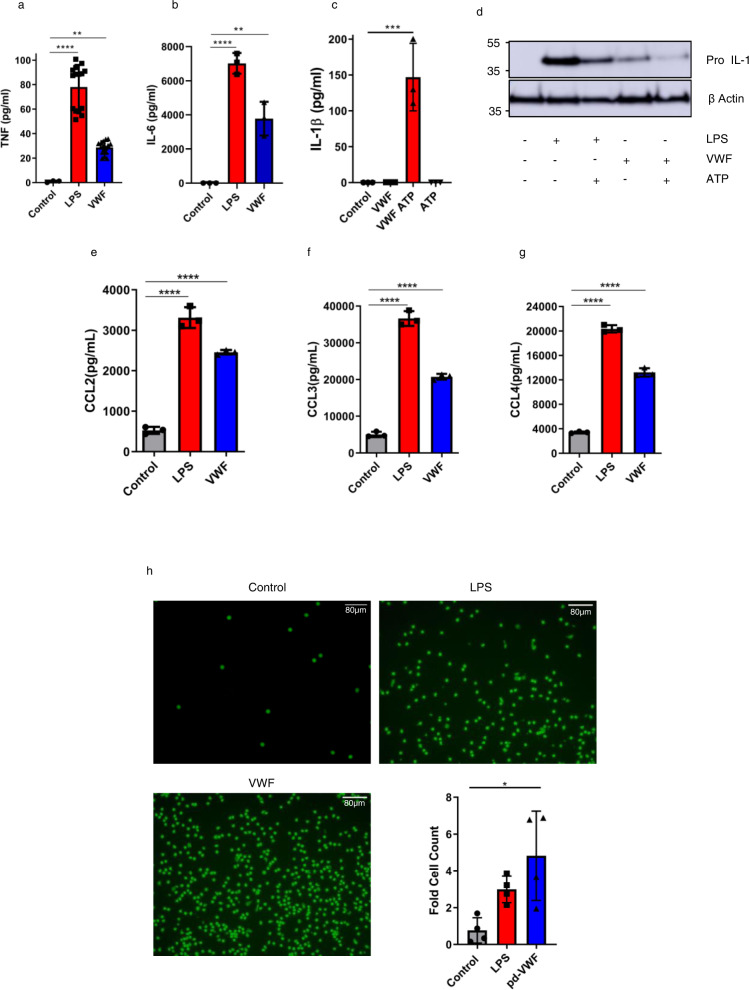
VWF induces pro-inflammatory cytokine and chemokine expression and promotes monocyte chemotaxis. **a** pd-VWF (10 µg/ml) or LPS (100 ng/ml) incubation with primary human macrophages for 4 h resulted in significant increases in secretion of **a** TNF (control vs LPS ****P < 0.0001, control vs VWF **P = 0.0052) and **b** IL-6 pro-inflammatory cytokines similar to LPS (control vs LPS ****P < 0.0001, control vs VWF **P = 0.0011). **c**, **d** Although VWF binding to macrophages was associated with an increase in IL-1β mRNA and intracellular pro-IL-1β levels, a significant increase in IL-1β secretion (and concurrent decrease in intracellular pro-IL-1β) levels was only observed when VWF-treated macrophages were also subsequently exposed to ATP as a second hit (***P = 0.0003). Similarly, pd-VWF (10 μg/ml) or LPS (100 ng/ml) stimulation of primary human macrophages for 24 h resulted in a significant increase in secretion of chemokines including (**e**) CCL2, **f** CCL3 and **g** CCL4 with ****P < 0.0001 control vs LPS and control vs VWF for all chemokines. All experiments were performed in triplicate, and the results shown represent the mean values ± standard deviation (SD) and the significance was determined by ANOVA respect to the control. *P < 0.05, **P < 0.01, ***P < 0.001 respectively. **h** Primary human macrophages were incubated with pd-VWF (10 μg/ml) or LPS (100 ng/ml) for 24 h. Cell supernatants were then collected and placed in the lower chamber of a transmigration wells. Naïve human monocytes were placed in the upper chamber and allowed to migrate for 2.5 h. Migrated cell numbers were stained with calcein-AM and assessed using Image J software. Monocyte migration induced by the supernatants from macrophages incubated with pd-VWF (10 μg/ml) (*P < 0.0207), LPS (100 ng/ml) (*P* = 0.213) or media are represented as average fold cell-count increase over four independent experiments and presented as mean values ± SD. pd-VWF (10 μg/ml) alone had no significant effect on the migration of naïve human monocytes (ns = not significant). All scale bars are 80 μM. Source data for this figure are provided as a Source Data file.

In keeping with the observed signaling effects, VWF binding to macrophages was associated with a significant increase in pro-inflammatory cytokine expression (including TNF and IL-6) (Fig. 3a, b). IL-1β protein secretion depends on NLRP3 inflammasome activation, which requires dual signal triggering^32^. Although VWF binding to macrophages was associated with an increase in IL-1β mRNA (Supplementary Fig. 3C), a significant increase in IL-1β secretion (Fig. 3c) and parallel decrease in intracellular pro-IL-1β concentrations (Fig. 3d) were only observed when VWF-treated macrophages were also subsequently exposed to ATP as a second stimulus demonstrating inflammasome activation by VWF. Control studies excluded endotoxin contamination of the pd-VWF product (Supplementary Fig. 6). Moreover, binding of the recently licensed clinical grade recombinant VWF (Vonvendi®, Takeda) to macrophages was also associated with proinflammatory signaling (Supplementary Fig. 7). Interestingly, the pro-inflammatory effects of clinical-grade rVWF were attenuated compared to pd-VWF, which may reflect differences in post-translational modification (in particular glycosylation) for rVWF compared to pd-VWF. Of note, the rVWF is expressed in Chinese hamster ovary (CHO-rVWF) cells which do not have the ability to generate α2–6 sialylation. Together, these findings demonstrate that VWF binding to macrophages directly initiates pro-inflammatory signaling, resulting in downstream pro-inflammatory cytokine production.

In view of the ability of VWF binding to upregulate pro-inflammatory cytokine secretion, we investigated its effects upon macrophage chemokine expression. VWF binding to primary human macrophages was associated with a significant increase in chemokine expression (including CCL2, CCL3, and CCL4) similar to that observed with the LPS-treated positive control (Fig. 3e–g). The potential functional significance of this VWF-induced chemokine expression was further investigated using a transmigration chemotaxis assay with supernatants collected from primary human macrophages stimulated with either pd-VWF, clinical-grade recombinant VWF or LPS respectively. Similar to LPS-treated positive controls cells, supernatants collected from macrophages stimulated with either pd-VWF or recombinant VWF were both effective in promoting enhanced monocyte transmigration (Fig. 3h and Supplementary Fig. 7B). Interestingly, however, the supernatant from both the pd- and recombinant VWF-treated macrophages was significantly more effective than LPS at recruiting monocytes (Fig. 3h and Supplementary Fig. 7B). Finally, we assessed whether VWF incubation influenced human macrophage phagocytic activity. VWF-treatment significantly (*p* < 0.05) attenuated macrophage phagocytosis of GFP-labeled *E. coli* in a manner similar to that observed with the LPS positive control. Collectively, these findings demonstrate that VWF binding plays a role in regulating macrophage cytokine and chemokine expression and thus has the potential to directly influence macrophage biology in vivo.

### VWF triggers macrophage polarization towards an M1 phenotype

**Fig. 4:**
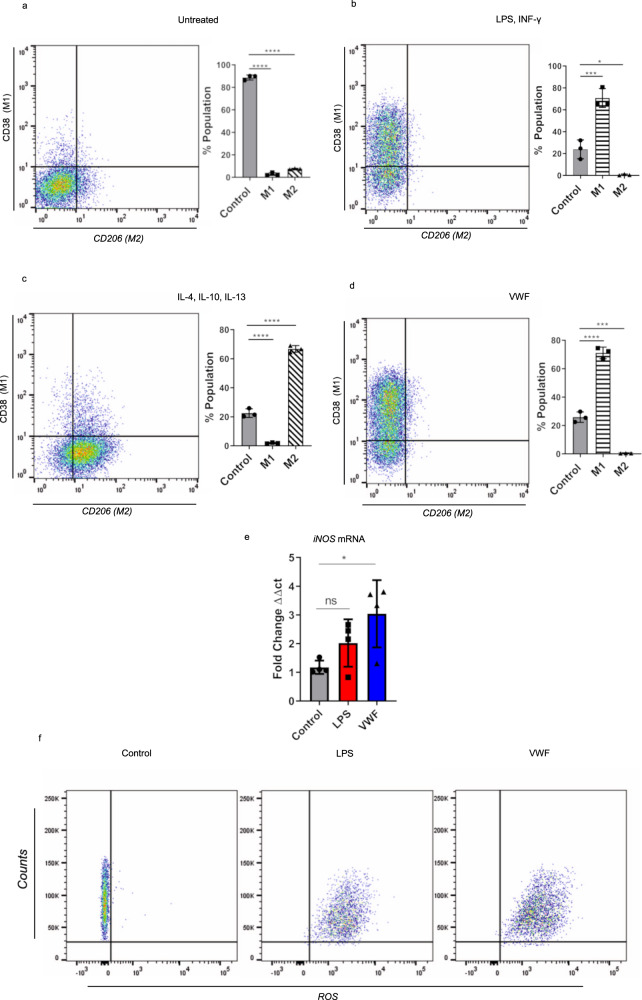
VWF triggers M1 macrophage phenotype. **a**–**d** Murine BMDMs were incubated in the presence or absence of a variety of different agonist combinations including (LPS 100 ng/ml and IFN-γ 20 ng/ml), (IL-4 40 ng/ml, IL-10 10 ng/ml, and IL-13 20 ng/ml) or pd-VWF (10 µg/ml) for 24 h and then cell surface marker expression was examined by flow cytometry. Flow gating strategy is presented in Supplementary Fig. 15. **a** Untreated control BMDMs expressed no CD38 or CD206. **b** The majority of BMDMS treated with LPS and INF-γ were CD38 positive, consistent with an M1 phenotype (*P = 0.0004). **c** In contrast, the majority of BMDMs incubated with IL-4, IL-10, and IL-13 were CD206 positive, consistent with an M2 phenotype (*P < 0.0001). **d** BMDM stimulation with pd-VWF (10 μg/ml) resulted in a significant increase in expression of CD38, consistent with a pro-inflammatory M1 macrophage phenotype (*P < 0.0001). Consistent with this M1 phenotype, **e** VWF treatment also resulted in a significant increase in iNOS expression in BMDMs (*P = 0.0287), **f** together with a marked increase in generation of reactive oxygen species (ROS). The data are presented as mean values ± SD for three independent experiments. The significance was calculated by ANOVA where *P < 0.05, **P < 0.01, ***P < 0.001 ****P < 0.0001; ns = not significant. Source data for this figure are provided as a Source Data file.

In view of the pro-inflammatory effects associated with pd- and recombinant VWF binding to macrophages, we investigated whether VWF interaction might influence macrophage polarization into M1 (classically activated or “pro-inflammatory”) or M2 (alternatively activated or “anti-inflammatory”) phenotypes. Incubation of murine BMDMs with LPS and IFN-γ resulted in ~75% of cells adopting an M1 phenotype (positive for C11b and CD38 expression) (Fig. 4a, b). In contrast, treatment with IL-4, IL-10, and IL-13 resulted in the majority of macrophages adopting an M2 phenotype (positive for CD11b and CD206) (Fig. 4c). Interestingly, treatment with VWF alone was sufficient to result in more than 70% of the BMDM adopting an M1 phenotype (Fig. 4d). Previous studies have demonstrated that generation of reaction oxygen species (ROS) and induction of nitric oxide synthetase (iNOS) constitute a hallmark feature of M1 macrophages^33,34^. We observed that VWF treatment of BMDM was associated with a significant increase in both iNOS expression (Fig. 4e) and ROS production (Fig. 4f). Together, these findings further support the hypothesis that VWF binding induces significant pro-inflammatory effects in macrophages.

### VWF regulates macrophage metabolism and drives glycolysis

**Fig. 5:**
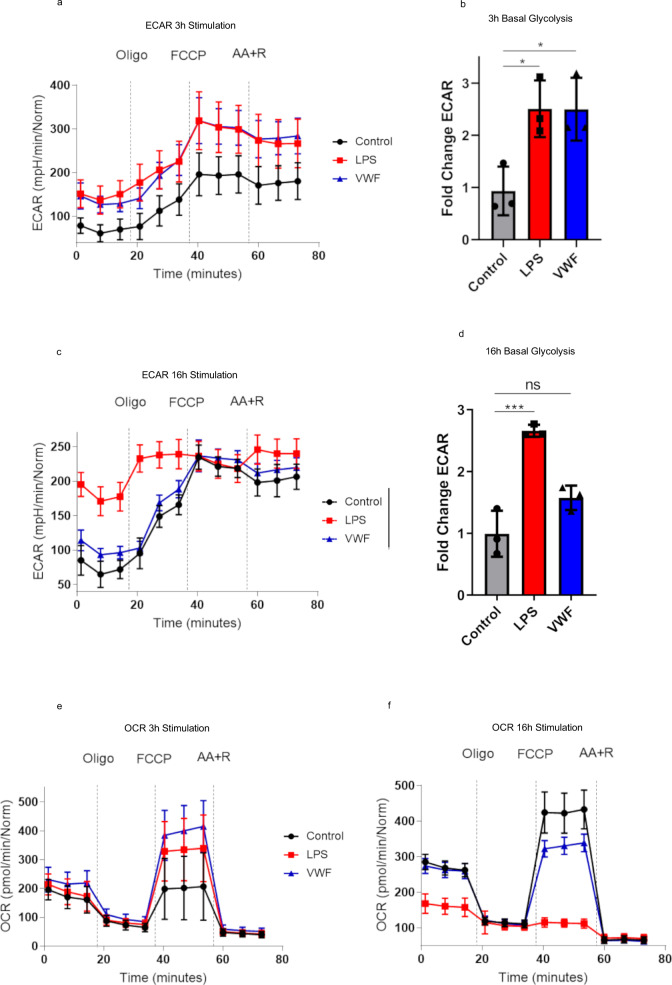
VWF regulates macrophage metabolism and drives glycolysis. Extracellular flux analysis (Seahorse XF Cell Mito Stress kit) was used to assess the effects of VWF-binding upon macrophage metabolism. Extracellular acidification (ECAR) was measured to study the effects on glycolysis following stimulation with VWF (10 μg/ml) (blue), LPS (100 ng/ml) (red), or untreated controls (black) for 3 h (**a**, **b**) and 16 h (**c**, **d**) respectively. Similarly, cellular oxygen consumption rate (OCR) was assayed to study the effects on BMDM oxidative phosphorylation after 3 h and 16 h (**e**, **f**) incubations. The effects of VWF and LPS on BMDM glycolysis (*P = 0.0272 and ***P = 0.0004 for control vs LPS; *P = 0.0279 and *P* = 0.0646 for control vs VWF, following 3 and 16 h respectively) and oxidative phosphorylation were studied in the presence or absence of specific mitochondrial inhibitors. Plots are representative images collected from three independent assays. The data are presented as the mean values ± SD for three independent experiments. Significance was determined by ANOVA in which **p < 0.01 and ****p < 0.0001. Source data for this figure are provided as a Source Data file.

Alterations in metabolic pathways, and in particular an increase in glycolysis, constitute a hallmark of inflammatory macrophages activated by both pathogen-associated and damage-associated signals through pattern-recognition receptor signaling^35,36^. To further investigate the hypothesis that VWF modulates macrophage function, the effects of VWF-binding on macrophage metabolism were assessed using extracellular flux analysis. Basal rates of glycolysis and oxidative phosphorylation were assessed using a Seahorse XF analyzer which measures extracellular acidification (ECAR) and cellular oxygen consumption rate (OCR) as readouts of glycolysis and mitochondrial respiration respectively. Basal rates of ECAR and OCR were assessed before and after the addition of mitochondrial inhibitors (including oligomycin, FCCP, or Antimycin A and rotenone (AA + R) respectively) as previously described^37^. Following 3 h stimulation with pd-VWF, a significant increase in ECAR (consistent with a marked increase in glycolysis) was observed in basal glycolysis (ECAR readings prior to the addition of Oligo) similar in magnitude to that observed with LPS exposure (Fig. 5a, b). Interestingly, and in keeping with the temporal effects of VWF observed on macrophage transcriptomics, after an extended 16-h incubation, the pd-VWF-induced increase in BMDM glycolysis had resolved, whereas ECAR remained significantly elevated in macrophages treated with LPS over the same time course (Fig. 5c, d).

OCR was used to assess mitochondrial oxidative phosphorylation (OXPHOS) after incubation with LPS or pd-VWF for 3 and 16 h. In contrast to its effect in promoting glycolysis, LPS or pd-VWF did not affect basal levels of mitochondrial oxidative phosphorylation (Fig. 5e). However, in keeping with previous reports^35,36^, we observed that 16 h exposure to LPS resulted in markedly reduced mitochondrial OCR (Fig. 5g). No such effect was observed in cells incubated with pd-VWF. Importantly, similar effects on macrophage metabolism were also observed when macrophages were treated with clinical-grade recombinant VWF in place of pd-VWF (Supplementary Fig. 7C). In addition, the effects of VWF on macrophage metabolism were not attributable to altered BMDM cell viability after stimulation (Supplementary Fig. 8A).

Transcriptomic analysis of VWF- or LPS-treated macrophages provided further insights into the mechanisms underlying these extracellular flux findings. In particular, we observed that VWF and LPS treatments induced distinct transcriptional changes in metabolic pathway genes at the 3 and 16 h time points (Supplementary Fig. 9). Importantly, transcription of key glycolysis, and TCA cycle -related genes in macrophages were specifically modified depending on the agonist (VWF or LPS), as well as the duration of exposure. While both VWF and LPS induced increased glycolysis at 3 h, we observed that these effects were driven by distinct changes in glycolysis-related gene expression between the two agonists. For example, VWF alone caused induction of Slc2a1 (a rate-limiting glucose transporter) at 3 h. Conversely at 3 h, LPS caused higher expression of Hexokinase II and III, the first enzymes in the glycolysis pathway. Similarly, genes involved in TCA cycle (including the isocitrate dehydrogase isozymes Idh1 and Idh2) were also differentially expressed depending on the agonist and duration of treatment.

### VWF influences mitochondrial morphology and upregulates HIF-1α expression

**Fig. 6:**
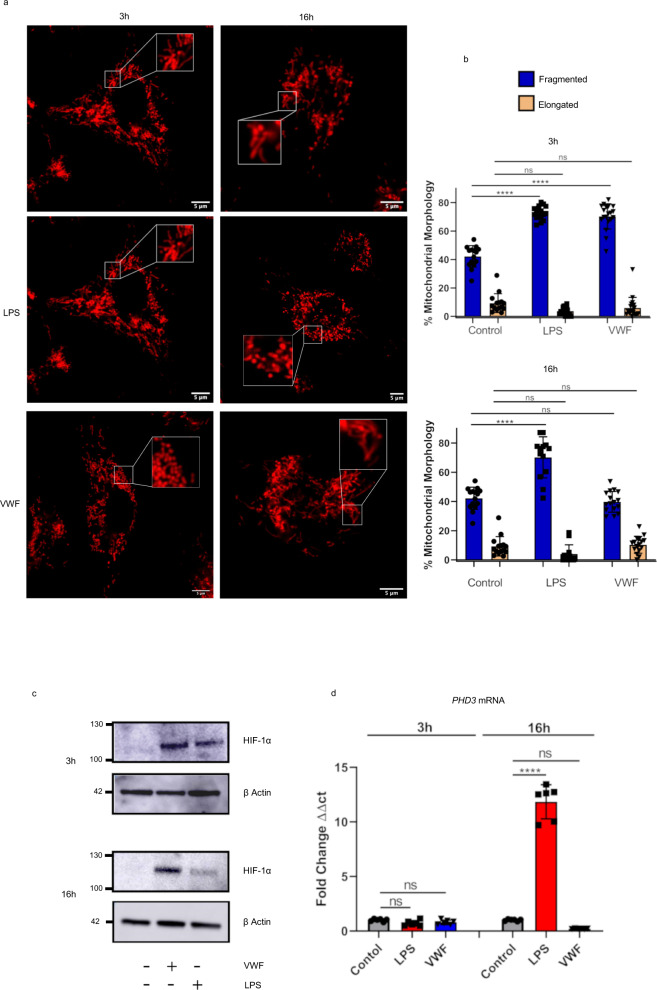
VWF influences mitochondrial dynamics and up-regulates HIF-1α expression. **a**, **b** Murine BMDMs were incubated in the presence or absence of pd-VWF (10 μg/ml) or LPS (100 ng/ml) for 3 or 16 h and mitochondrial morphology assessed using Mitotracker and scanning confocal live cell imaging as detailed in the Materials and Methods. A minimum of 20 images including ≥60 mitochondria per cell were analyzed per treatment. Following 3 h stimulation with either VWF or LPS, a significant increase in mitochondrial fragmentation consistent with an increase in glycolysis was observed (*p* < 0.01 and *p* < 0.001 respectively). Although a significant increase in mitochondrial fragmentation was still observed following a 16 h incubation with LPS (****P < 0.0001), it was no longer observed in BMDMs treated with VWF (*P* = 0.958, ns = not significant). **c**, **d** BMDMs were treated with either VWF (10 μg/ml) or LPS (100 ng/ml) for 3 or 16 h (****P < 0.0001 for control vs LPS and *P* = 0.3065 for control vs VWF at 16 h), and then HIF-1α or PHD3 expression were assessed using Western blotting or qRT-PCR respectively. The data are presented as mean values ± SD for three independent experiments. The significance was calculated by ANOVA where *P < 0.05, **P < 0.01, ***P < 0.001, ****P < 0.0001 respectively. Source data for this figure are provided as a Source Data file.

Previous studies have reported that changes in mitochondrial morphology accompany changes in macrophage metabolic state^38^. In particular, mitochondria adopt a fragmented appearance when glycolysis activity is high, as opposed to an elongated state during periods of heightened oxidative phosphorylation^38–40^. In keeping with this hypothesis, we observed that LPS stimulation of macrophages for either 3 or 16 h resulted in fragmented mitochondrial morphology (Fig. 6a, b). Interestingly, treatment with VWF for 3 h was also associated with significantly higher levels of mitochondrial fragmentation. However, in contrast to LPS, mitochondrial morphology had returned to normal following 16 h exposure to VWF. These findings further support the hypothesis that VWF binding has significant modulatory effects upon macrophage mitochondrial morphology, which will impact on mitochondrial metabolism and, in particular, demonstrate that VWF promotes short-term marked increases in macrophage glycolysis.

The ability of LPS to promote macrophage glycolysis, even with prolonged exposure, has been attributed at least in part to an upregulation in HIF-1α expression^41,42^. To investigate potential mechanisms through which VWF promotes macrophage glycolysis in a time-dependent manner, HIF-1α expression following 3- and 16-h incubations with VWF or LPS respectively was assessed. After 3-h incubation with either VWF or LPS, a significant increase in macrophage HIF-1α protein expression levels was observed (Fig. 6c). Interestingly, although HIF-1α expression remained elevated in BMDMs after a 16-h incubation with LPS, levels were reduced in VWF-treated cells following this extended treatment (Fig. 6c). Furthermore, we observed similar time-dependent effects of LPS and VWF on the expression of macrophage PHD3 which is a key negative regulator of HIF-1α expression (Fig. 6d)^43^.

### Macrophage LRP1 plays a critical role in regulating VWF inflammatory signaling

**Fig. 7:**
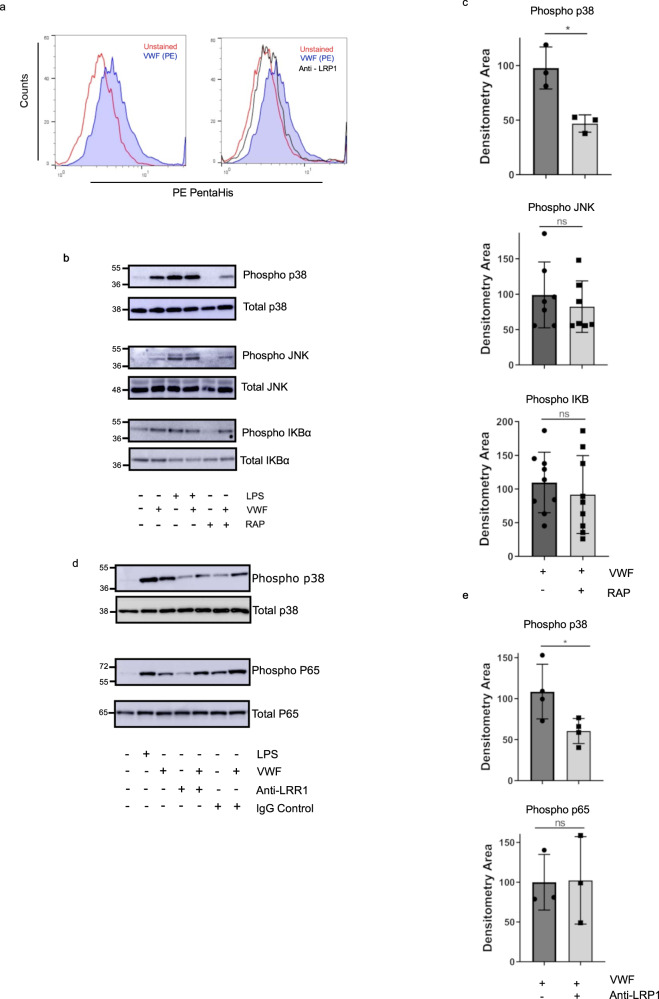
LRP1 plays a crucial role regulating VWF inflammatory signaling. **a** Flow cytometry was used to determine the binding of pd-VWF to human primary macrophages in the presence of LRP1 blocking antibody. Flow gating strategy is presented in Supplementary Fig. 14. Representative histogram of VWF bound to macrophage (blue) and pd-VWF bound to macrophage in the presence of anti-LRP1 antibody (black). **b** Inhibition of human macrophage LRP1 binding with receptor associated protein (RAP) decreased p38 phosphorylation by 50% (*P = 0.013), with no alteration in JNK or NF-κB activation (ns = not significant, *P* = 0.642 and *P* = 0.374, respectively). **c** Densitometry of western blots determined from 3 independent experiments. **d** Inhibition of LRP1 using a monoclonal anti-LRP1 antibody resulted in similar effects as RAP inhibition with p38 signaling reduced 50% (*P = 0.0394), and no alteration in NK-κB activation (*P* = 0.953). **e** Densitometry of western blots determined from three independent experiments (mean ± SD). The significance was determined by Mann Whitney or t-test in which *P < 0.05. Source data for this figure are provided as a Source Data file.

Recent studies have reported that the LRP1 receptor on both human and murine macrophages can bind to VWF, and that it plays a role in regulating VWF clearance in vivo^25,44–46^. In keeping with those data, we confirmed that VWF binding to primary human macrophages was attenuated in the presence of anti-LRP1 antibody (Fig. 7a). Binding of a number of other LRP1 ligands have been reported to initiate intracellular signaling in macrophages^47,48^. To investigate whether VWF-LRP1 binding is involved in triggering VWF pro-inflammatory signaling, primary macrophages were incubated with pd-VWF (10 μg/ml) in the presence or absence of RAP (200 nM). In the presence of RAP, a significant reduction in VWF-induced MAPKinase p38 phosphorylation was observed (Fig. 7b, c). Consistent with previous studies demonstrating that LPS binding to TLR4 leads to activation of JNK, p-38, and NF-κB activation^49^, RAP did not inhibit LPS-induced p38 phosphorylation (Supplementary Fig. 8C). Moreover, RAP did not attenuate VWF-induced JNK or NF-κB activation (Fig. 7b, c). Consistent with the RAP data, anti-LRP1 inhibitory antibody also significantly reduced VWF-induced p38 signaling but did not alter NF-κB signaling determined by p65 (Fig. 7d, e). Together, these findings demonstrate that VWF interaction with macrophages drives activation of the p38 MAPKinase pathway, which is modulated at least in part through the LRP1 receptor.

**Fig. 8:**
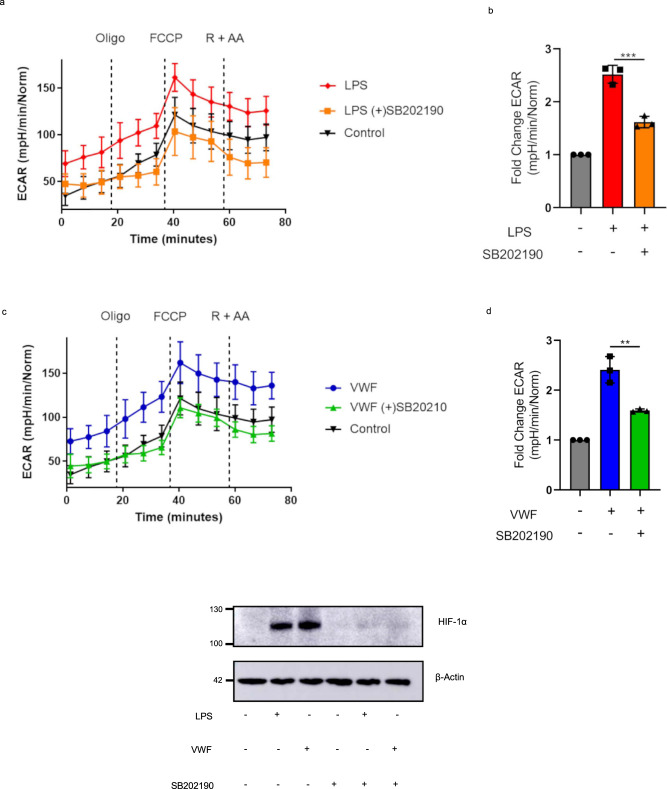
p38 and HIF-1α triggered enhanced glycolysis produced following VWF stimulation. Representative plots of BMDMs extracellular acidification rate (ECAR) and bar charts of fold change of the basal ECAR following 3 h treatment and with **a**, **b** LPS (100 ng/ml) or **c**, **d** VWF (10 μg/ml) with or without 1 h pre-treatment of p38 inhibitor, SB202190 (50 μM). Fold change ECAR was determined from three independent experiments ± SEM. Significance was determined by ANOVA (**p < 0.01). **e** BMDMs were treated for 3 h with pd-VWF (10 μg/ml) or LPS (100 ng/ml) with or without SB202190 (50 μM) 1 h pre-treatment. HIF-1α levels then examined by Western blot and total protein determined by β-actin. All experiments were performed in triplicate and source data for this figure are provided as a Source Data file.

We further investigated whether MAPKinase p38 might play a role in regulating VWF-dependent effects on macrophage glycolysis. Importantly, the p38 MAPKinase inhibitor SB202190 significantly reduced the ability of LPS (Fig. 8a, b) or VWF (Fig. 8c, d) to promote macrophage glycolysis but did not affect cell viability (Supplementary Fig. 8B). Furthermore, this was confirmed to be HIF-1α dependent, as p38 inhibition also prevented VWF-induced downstream HIF-1α activation (Fig. 8e). Cumulatively these findings demonstrate that the proinflammatory effects in macrophages triggered by VWF interaction with LRP1 are mediated through p38 activation, which in turn causes early stabilization of HIF-1α and enhanced glycolysis.

In keeping with previous studies^25,46^, we observed that multiple domains within VWF contribute to macrophage binding (Supplementary Fig. 10A–E). Initial experiments demonstrated that N-terminal VWF-D’A3 and the C-terminal VWF-A3CK truncations could both bind to macrophages (Supplementary Fig. 10B–C). The binding of full-length VWF and VWF truncations (VWF-A1A2A3 and VWF-D’A3) to macrophages was significantly enhanced in the presence of ristocetin which causes A1 domain unfolding similar to that induced by shear-stress (Supplementary Fig. 10B, D). Finally, we assessed macrophage binding for individual A1, A2, and A3 domains respectively. Significant binding was observed for the A1 domain, but not for either the A2 or A3 domains (Supplementary Fig. 10E). Similarly, we observed that multiple VWF domains (including VWF-D’A3, VWF-A1A2A3, and VWF-A1) could bind to immobilized LRP1 and that binding was enhanced in the presence of ristocetin. Collectively, these findings highlight a key role for the A1 domain in modulating macrophage and LRP1 interaction, but further confirm that other N- and C-terminal domains of VWF can also interact with macrophages. This hypothesis is supported by the observation that an anti-VWF antibody targeted to the A1 domain can attenuate but not completely inhibit VWF binding to macrophages under shear stress conditions (Supplementary Fig. 11).

### VWF has pro-inflammatory and chemo-attractive effects in vivo

**Fig. 9:**
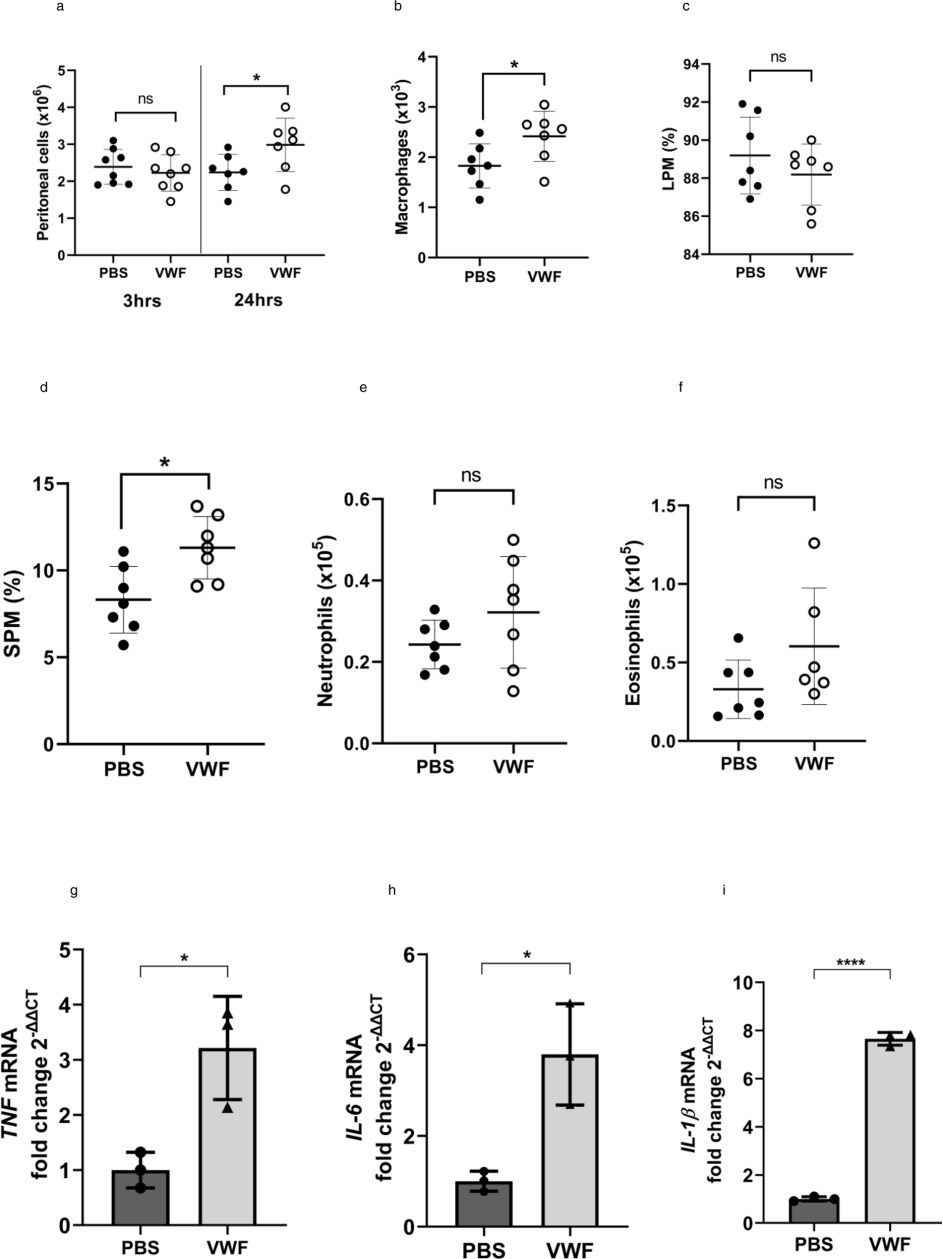
Intraperitoneal injection of VWF activates macrophages in the peritoneal cavity of mice. Mice were injected IP with VWF (2 mg/kg) or PBS (control). After 3 or 24 h post-injection periods, mice were sacrificed, peritoneal lavage performed and peritoneal exudate cells (PEC) recovered and analyzed by flow cytometry (see Supplementary Fig. 10) and gene expression analyzed by qPCR. **a** Total numbers of PEC cells recovered from mice (*P* = 0.508 and *P = 0.0428 for 3 and 24 h respectively). **b** Numbers of macrophages present in PEC of mice (*P = 0.0383) and relative frequency (%) of **c** large peritoneal macrophages (LPM) (*P* = 0.320 and **d** small peritoneal macrophages (SPM) (*P = 0.0108). Numbers of **e** neutrophils (*P* = 0.187) and **f** eosinophils in PEC (*P* = 0.112). Data are presented as mean ± SD from 7 to 8 mice per group and from two biological replicates. **g**, **h** To validate the changes detected by flow cytometry in peritoneal macrophages after VWF treatment in vivo, RNA was isolated from PEC before and after treatment with VWF or PBS respectively. Consistent with our RNAseq data and in vitro studies, VWF-treatment was associated with significant increases in **g** TNF (***P = 0.0004), **h** IL-6 (**P = 0.0061), and **i** IL-1β (****P < 0.0001) expression were observed in PEC at 3 h. Statistical significance was determined using *t*-test in which *p < 0.05, **p < 0.01, ***p < 0.001. Source data for this figure are provided as a Source Data file.

In vitro binding of VWF to macrophages is associated with polarization towards an M1 pro-inflammatory phenotype, metabolic changes and production of pro-inflammatory cytokines and chemokines. To further investigate the potential in vivo significance of these observations we utilized a previously described model of chemotaxis in which clinical-grade rVWF or saline control were injected into the peritoneum of wild-type mice^50–52^. Peritoneal lavage was then performed, and peritoneal exudate cells (PEC) recovered for analysis by flow cytometry (Supplementary Fig. 12). Three hours after intraperitoneal injection of clinical-grade VWF into mice, there was a non-significant reduction in the total number of cells recovered from the peritoneum with no changes in the cellular composition of the PEC compared to PBS-injected control mice (Fig. 9a). In contrast, after 24 h there was a significant (*P* < 0.05) increase in numbers of PEC recovered in VWF-injected mice relative to PBS-injected mice (Fig. 9a). Flow cytometry phenotyping of PEC cells demonstrated that clinical-grade VWF elicited a significant (*P* < 0.05) increase in macrophages (CD45 + CD11b + Siglec-Ly6G-) in the peritoneum (Fig. 9b). There are two macrophages (CD45 + CD11b + Siglec-Ly6G-) subsets in the peritoneum of mice. Large peritoneal resident macrophage (LPM) populations (F4/80hiMHCIIlo) constitute the main population (~80–90%) of peritoneal macrophages in naive mice under steady state conditions. In contrast, small peritoneal macrophage (SPM; F4/80lowMHCIIhi) represent a minor (~10%) population that expands in response to inflammatory stimuli. Injection of VWF did not significantly alter the resident LPM population, but caused a significant increase (*P* < 0.05) in SPM (Fig. 9c, d). In contrast, clinical-grade VWF did not induce any significant changes in neutrophils or eosinophils within the peritoneum (Fig. 9e, f). To further validate the changes detected by flow cytometry in peritoneal macrophages after VWF treatment in vivo, RNA was isolated from PEC before and after treatment with VWF or PBS respectively. Consistent with our previous RNAseq data and in vitro studies, VWF-treatment was associated with significant increases in TNF, IL-6, and IL-1β expression in PEC at 3 h (Fig. 9g–i). Collectively, these in vivo data support the hypothesis that macrophages represent a primary target for the pro-inflammatory response induced by VWF.

## Discussion

Accumulating recent data has demonstrated that VWF not only regulates primary hemostasis, but also has direct effects upon inflammatory responses^10,12–16^. These pro-inflammatory properties of VWF have been observed in a variety of different murine inflammatory disease models and have been independently validated in experiments using either VWF-blocking antibodies or VWF^−/−^ mice^12–14^. However, the biological mechanisms underpinning the immuno-modulatory effects of VWF remain poorly understood. In vitro studies have confirmed that immobilized VWF can bind directly to leukocytes^31,53^. Under flow conditions, this VWF-leucocyte interaction was shown to consist of initial transient rolling (mediated via VWF binding to leukocyte PSGL-1) followed by stable adhesion (mediated by VWF interaction binding to leukocyte β2-integrins)^31^. In keeping with previous studies demonstrating a role for liver Kupffer cells in regulating VWF clearance^24,26^, we observed binding of both pd-VWF and recombinant VWF to primary human macrophages, THP-1 macrophages, and murine BMDMs respectively. As reported previously, however, no significant VWF binding to undifferentiated primary monocytes was seen^31^. Based on these findings, we propose that VWF and monocytes circulate together in normal blood with minimal interaction. However, at sites of vascular injury, VWF comes into contact with tissue-resident macrophages. VWF will bind to these macrophages because they express a different repertoire of surface receptors. Importantly, our data further highlight that VWF does not simply bind to macrophages. Rather, VWF binding also initiates significant downstream signaling effects, including phosphorylation of the MAPKinase pro-inflammatory pathway p38 and JNK in addition to NF-κB activation. Although VWF-induced signaling in macrophages has not previously been described, our findings are consistent with prior studies demonstrating that VWF binding to the glycoprotein (GP) Ib-IX-V receptor on platelets results in complex intracellular signaling that involves a number of different intracellular molecules including the Src family, Rac1, PI3-kinase/Akt, and MAP kinases^54–56^.

In keeping with the pro-inflammatory signaling associated with VWF binding to primary human macrophages or murine BMDMs, we observed significant increases in (i) proinflammatory cytokine expression (ii) chemokine expression (iii) iNOS expression and ROS production. Furthermore, VWF binding induced the majority of macrophages to adopt an M1 inflammatory phenotype. Unsurprisingly, given these major effects on macrophage biology, using extracellular flux analysis we observed that VWF also had significant effects upon macrophage metabolism and in particular glycolysis. A short incubation with either pd- or recombinant VWF resulted in a marked initial increase in macrophage glycolysis similar to that observed with LPS. Glycolysis was still elevated with prolonged exposure of LPS, however, increased glycolysis was not sustained under longer exposure of VWF. Interestingly the suppression of oxidative phosphorylation observed with prolonged LPS stimulation, did not occur with VWF. In agreement with other reports^38,39^, LPS stimulation led to an increase in mitochondrial fission, and a similar increase in fission was observed with short incubation of VWF. Enhanced fission observed with VWF is likely contributing to the higher mitochondrial ROS in these cells and the promotion of the pro-inflammatory phenotype. Similarly, a significant increase in macrophage HIF-1α expression levels (which are known to play an important role in promoting macrophage glycolysis^41,42^) was observed following a 3-h incubation with VWF, but had normalized by 16-h. Our data further demonstrate that MAPKinase p38 is involved in the activation of rapid HIF-1α dependent glycolysis following VWF treatment. Collectively, these data support the hypothesis that VWF binding has significant but short-term pro-inflammatory effects on macrophage biology.

Previous studies have highlighted a number of important clinical circumstances when VWF comes into contact with macrophages. In particular, following tissue damage and blood vessel injury, VWF escapes from the plasma into subendothelium where it comes into contact with tissue-resident macrophages. Based upon our findings, it is clear that VWF-binding will trigger these macrophages to adopt an M1 phenotype, with the consequent secretion of proinflammatory cytokines and chemokines at the site of vascular damage, and recruitment of further macrophages to the site of injury. Thus, our data highlight that VWF not only plays a key role in initiating primary hemostasis and platelet plug formation at the site of blood vessel injury, but also that it can plays a role in innate immunity by priming macrophages in the vicinity to promote a pro-inflammatory response.

The proinflammatory effect of VWF-binding on macrophage biology described herein has additional important implications beyond sites of vascular injury. Previous meta-analyses and systematic reviews have reported that elevated levels of VWF are associated with increased risk for ischemic heart disease (IHD)^57–59^. Conversely, patients with von Willebrand disease appear to be relatively protected from IHD development^60,61^. Furthermore, ABO blood group has also been shown to constitute a risk factor for cardiovascular disease, with significantly reduced cardiovascular risk in group O compared to non-O (A, B or AB) individuals. Of note, plasma VWF levels are 20–30% lower in blood group O compared to non-O subjects^62–64^. Interestingly, studies performed in VWF^−/−^LDLR^−/−^ double-knockout mice demonstrated significantly reduced atherosclerotic plaque formation compared to VWF^+/+^LDLR^−/−^ controls^65^. Importantly, a marked reduction in macrophage accumulation within atheromatous plaques was also observed in VWF^−/−^ mice^65^. In addition, recent studies performed using ADAMTS13^−/−^ mice further support the hypothesis that VWF is important in IHD pathogenesis. Once again, macrophage recruitment into atheromatous plaque lesions was significantly increased in ApoE^−/−^/ADAMTS13^−/−^ compared to ApoE^−/−^ /ADAMTS13^+/+^ controls^66^. Cumulatively, these findings support the hypothesis that VWF plays a role in modulating macrophage recruitment into atheromatous plaques.

Recent studies have reported that a number of macrophage receptors can modulate VWF binding^26^. Our data demonstrate that VWF binding to LRP1 plays a specific role in triggering pro-inflammatory signaling in macrophages. Thus, inhibition of LRP1-binding using either RAP or anti-LRP1 antibodies significantly attenuated the ability of VWF to drive MAPKinase p38 phosphorylation and subsequent HIF-1α activation (Supplementary Fig. 13 - Graphical Abstract). In contrast, LRP1 inhibition failed to attenuate VWF-induced JNK or NF-κB activation. Together, these data demonstrate a role for LRP1 in driving VWF-dependent macrophage activation, but further suggest that additional macrophage receptors are also involved. Interestingly, a role for tissue-type plasminogen activator (tPA) binding to LRP1 in regulating macrophage activation has also been proposed^47,48^. However, the normal plasma concentration of VWF is ~10 µg/ml, as opposed to <20 ng/ml for tPA. From the VWF aspect, several different domains of VWF have been implicated in regulating LRP1 binding^45,46^. In particular, we have recently described a key role of the VWF A1 domain in this context^25^. Since VWF circulates as a series of heterogeneous multimers, the potential for complex interactions on the macrophage surface are readily apparent. Further studies will important to characterize other macrophage receptors involved in modulating the pro-inflammatory signaling effects of VWF, and to investigate potential synergistic binding effects between different receptors.

In conclusion, recent studies have described the complex cross-talk that exists in vivo between hemostasis and inflammation, and developed the concept of immuno-thrombosis^67–69^. Our data define a biological role and mechanism for VWF in driving inflammatory responses, and thereby establish another link between primary hemostasis and innate immunity. Thus, VWF not only plays a key role in the initiation of hemostasis at sites of vascular injury, but also functions to prime local macrophages to initiate pro-inflammatory responses. In this local milieu, we propose that VWF functions as a damage signal that is recognized through specific macrophage pattern-recognition receptors. In addition, our findings also provide insights into the effects of VWF binding on macrophage biology that may help to explain the accumulating evidence that VWF is involved in the pathogenesis of a number of different murine inflammatory disease models. Given the significant morbidity and mortality associated with inflammatory pathology, defining the roles of VWF in this context may offer exciting opportunities to develop novel therapies to target these pathways and address an important unmet clinical need.

## Methods

This study was approved by the St James’ Hospital Research Ethics Committee (2017/01/6) and the Irish Blood Transfusion Society (IBTS-004-03-18). Written informed consent was provided by all healthy blood donor participants. All murine experiments were approved by the Royal College of Surgeons in Ireland Ethics Committee (REC1315) and performed on mice 6–8 weeks old, in full accordance with the Health Product Regulatory Authority, Ireland (AE19136/P040).

### Reagents

Human plasma-derived von Willebrand factor-Factor VIII free (VWF) (Haematology Technologies Inc.), Recombinant von Willebrand factor, VonVendi® (r-VWF) (Takeda), Ultra pure lipopolysaccharide (LPS) (Sigma), INFγ (Life Technologies, Gibco), IL-4 (Life Technologies, Gibco), IL-10 (Life Technologies, Gibco), IL-13 (Life Technologies, Gibco). Recombinant mouse M-CSF (rm M-CSF) (R&D System), Cell Tracker green CMFDA Dye (Thermofisher), Seahorse XF Cell Mito Stress Cell Kit (Agilent), Primary antibodies human anti-p38, P-p38, JNK, P-JNK, IKBα, P-IKBα, p65, P-p65, HIF-1α, and β-actin (Cell Signaling Technologies), Alexa Fluor 488 conjugate (Molecular Probes, Thermofisher Scientific).

### Cell culture

Peripheral blood mononuclear cells (PBMC) were isolated from health donor buffy coats following histopaque (Sigma) gradient separation. Anti-CD14 beads (Miltenyi Biotec) were used to isolate monocytes. Isolated monocytes were differentiated into macrophages for 7–10 days in the RPMI media supplemented with 10% human serum (Sigma), Penicillin-Streptomycin 100 µg/ml (Life Technologies, Gibco). Murine PBMC were isolated from the bone marrow from 8–12 weeks old C57/B6JB mice. PBMC were incubated for 7 days in RPMI supplemented with 10% fetal bovine serum (Life Technologies, Gibco), 25 ng/ml rmM-CSF and Penicillin-Streptomycin 100ug/ml to generate bone marrow-derived macrophages (BMDM). THP1 monocytes were differentiated into macrophages in the presence of 100 nM PMA (Sigma) for 3 days.

### Western blotting

Cells were lysed in RIPA Buffer (ThermoFisher) supplemented with protease (Merck) and phosphatase (Sigma) inhibitors and normalized using BCA Protein Assay Kit (Pierce, ThermoFisher). Samples were resolved by SDS-PAGE. Primary antibodies were incubated overnight at 4 °C and subsequently incubated with IgG-HRP antibodies. Blots were developed using chemiluminescence staining (ECL, Pierce, Thermofisher).

### mRNA isolation and qRT-PCR

Total mRNA was isolated using Trizol (Sigma). cDNA was synthesized using RevertAid reverse transcriptase (ThermoFisher) and RT-qPCR was performed in triplicates with Go Taq qPCR master mix (Promega) using Life Technologies 7500 Real-Time PCR System. The mRNA level was normalized to β-actin. To determine the activation of PHD3 and iNOS cell lysates and RNA were isolated from human primary macrophages after 24 h treatment of VWF or LPS. Primer sequences are listed in Supplementary Table 1.

### Cytokine analysis

Human primary macrophage IL-1β, TNFα, and IL-6 cytokines were quantified by ELISA (Invitrogen) following a 4 or 24 h treatment with either 10 µg/ml of VWF or 100 ng/ml of LPS in RPMI supplemented with 1 mM CaCl_2_. Human primary macrophage inflammasome activation was detected after 24 h incubation with VWF or LPS. Cells were subsequently incubated with ATP 5 mM for 1 h in serum free RPMI and pro-IL1β was determined by western blot (goat anti-pro-IL-1β CST).

### RNA sequencing

BMDM were incubated overnight in RPMI GlutMax supplemented with 10% FBS, penicillin (100 IU/ml), streptomycin (100ug/ml) and recombinant murine (M-CSF, 25 μg/ml). Cells were washed and then incubated with pd-VWF (10 μg/ml) or LPS (100 ng/ml) for 3.5 h (T1) or 16 h (T2) respectively. Supernatant was removed and BMDM were washed with PBS. RNA was extracted with the RNeasy Kit (QIAGEN) and samples frozen at −80 °C. RNA sequencing analysis was performed by Novogene® using a NovaSeq 6000 S4 platform. Treated BMDM were compared to unstimulated BMDM controls.

### RNAseq data analysis

Paired-end reads of 150 bp length were mapped against mouse assembly GRC38 (mm10) and quantified using the short read mapper STAR^70^. Removal of lowly expressed genes was based on TPM values (transcripts per million). Genes that stayed below 10 TPM in all samples that were used in a comparison were excluded. Differential expression analysis was carried out with DESeq2^71^. For principle component analysis, data were transformed using regularized log transformation with DESeq2. PCA was performed using the prcomp function in R and using all genes that passed TPM cutoff (*n* = 8737). Multiple testing correction of p-values was carried out using the Benjamini-Hochberg method. Differentially expressed genes were identified using an FDR < 0.05 and |log2 foldchange| >  1. Heatmaps and volcano plots were generated in R with the packages ComplexHeatmap (version 2.12.0) and ggplot2 (version 3.3.6).

### VWF binding to human and murine macrophages

To evaluate VWF binding to macrophages by flow cytometry, human monocyte-derived macrophages were incubated with pd- or recombinant VWF in RPMI + 1 mM CaCl_2_ for 30 min on ice. Fc receptors were blocked using a Fc-gamma receptor inhibitor (Thermofisher). Bound VWF was detected using polyclonal rabbit anti-human VWF (Dako, Agilent) for 30 min followed by anti-rabbit Alexa-488 (Thermofisher) for 30 min. Bound recombinant VWF was detected using a PE-labeled Anti-His Tag antibody (BioLegand).

For confocal microscopy, monocytes were differentiated on glass coverslips (Nunc, Lab-Tek). Macrophages were incubated with 10 µg/ml of VWF at room temperature in RPMI supplemented with CaCl_2_ 1 mM for 30 min. Cells were blocked with Fc-gamma receptor inhibitor (ThermoFisher) and 3% BSA and incubated with anti-VWF and anti-rabbit Alexa 488. THP1 cells were differentiated and incubated with anti-VWF antibody and anti-early endosomal antigen 1 (EEA1) antibody (Santa-Cruz). The cell membrane was labeled with cell mask deep red (Molecular probes, Thermofisher Scientific) cells were mounted with mounting media and in-situ DAPI stain (Sigma).

To further characterize the role of specific VWF domains in modulating interaction with macrophages and the LRP1 receptor, a series of recombinant VWF truncations were expressed and purified as before^25^. Briefly, the expression vector pcDNA-VWF encoding full-length rVWF has previously been described^25^. A DNA fragment containing VWFA1A2A3 (residues 1260 to 1874) was inserted into expression vector pEXPR-IBA 42 (IBA, Germany) via NheI and PmeI restriction sites. The same strategy was used for expressing VWFA1 (residues 1239–1472), VWFA2 (residues 1473–1668) & VWFA3 (residues 1671–1878). Human full-length recombinant VWF (rVWF), VWF-D’A3 VWF-A3CK, VWF-A1A2A3, VWF-A1, VWF-A2, and VWF-A3 were then all transiently expressed in HEK293T cells. Conditioned serum free medium was harvested, concentrated, and further purified via nickel affinity chromatography^25^. Subsequently, the binding of VWF truncations to BMDM and THP-1 macrophages was assessed using flow cytometry. Where indicated binding studies were performed in the presence or absence of ristocetin (1.5 mg/ml).

To examine VWF interaction with macrophages under shear, THP-1 cells were resuspended in differentiation media (serum-free RPMI media, 0.1% BSA, 1 mM MnCl_2_ and 100 nM PMA) for 15 min at 37 °C. Differentiated THP-1 cells were then fluorescently labeled with CMFDA-cell tracker green (Invitrogen) at 2.5 µM for 30 min at 37 °C. pd-VWF in Protein Free Blocking solution (PFBS) (Thermo Fisher) was coated onto μ-slides VI 0.4 (Ibidi GmbH, Germany) overnight. Non-coated control channels were blocked with PFBS. Laminar shear flow was induced using a syringe pump (Nemesys S, Cetoni GmbH, Germany). Each lane of the slide was connected to the pump and differentiated THP-1 cells perfused across the channels at 0.25 ml/min corresponding to a venous wall shear rate of 0.31 dyn cm^−2^. VWF-adhered THP-1 cells were observed in real-time using an EM-CCD camera (Andor iXon 888) on a Zeiss Axiovert microscope controlled by Metamorph v7.8.2 software. Time-lapse image sequences were acquired at 17 frames per second using a 10x Plan Neofluar (NA 0.3) objective. Subsequently, time-lapse image sequences were analyzed. In brief, fluorescently labeled THP-1 cells were detected in each frame. The xy location and area of the cells were passed on to the particle tracking software uTrack and tracks with a length of <3 frames were discarded. A global fit was performed simultaneously to the cumulative number of tracks over time to obtain binding rates (number of cells mm^−2^ s^−1^) and detachment rates (s^−1^). The equilibrium surface coverage was calculated by dividing the binding rate by the detachment rate. Where indicated, shear perfusion experiments were performed in the presence of polyclonal anti-VWF or anti-A1 domain antibodies (20 µg/ml).

### Role of VWF in Macrophage Polarization

Murine - Bone marrow-derived macrophages (BMDM) were cultured with rmM-CSF (25 ng/ml) for 24 h. In addition, LPS (100 ng/ml) and IFNγ (20 ng/ml) were added to generate a M1 polarized phenotype. IL-4 (40 ng/ml), IL-13 (20 ng/ml) and IL-10 (10 ng/ml) was added to generate M2 polarized macrophages. Alternatively, VWF (10 μg/ml) in RPMI with 1 mM CaCl_2_ was used. Cells were examined for surface marker expression by flow cytometry. M1 macrophages were dual positive for CD11b (BioLegend) and CD38 (BioLegend). M2 were dual positive for CD11b and CD206 (BioLegend). Cellular reactive oxygen spices (ROS) generation was detected using CellROX DeepRed staining (ThermoFisher). BMDM were incubated with VWF or LPS for 3 h and analyzed by flow cytometry after exclusion of dead cells by Live-Dead FITC (ThermoFisher).

### Measurement of VWF-mediated monocyte chemotaxis and transmigration

BMDM were stimulated with VWF (10 μg/ml) or LPS (100 ng/ml) in RPMI supplemented with CaCl_2_ (1 mM) and M-CSF (25 ng/ml) for 24 h. Supernatants were harvested and placed in the lower chamber and isolated human naive monocytes placed in the top chamber. Monocytes were allowed to migrate to the lower chamber for 2.5 h. Migrated cells were stained using Cell Tracker green (ThermoFisher) for 30 min. Cell counts were quantified using ImageJ software and represented as fold change from control.

### Effects of VWF on macrophage metabolism

Following BMDM differentiation, cells were seeded into Seahorse XF96 culture plates (Agilent) at a density of 5 × 10^5^ well and were stimulated with LPS (100 ng/ml) or VWF (10 μg/ml) for 3 h or 16 h in RPMI supplemented with 1 mM CaCl_2_. Following treatment BMDM were incubated with Seahorse phenol red-free base media in a CO_2_ free incubator as per manufacturer’s instructions (Agilent). To determine extracellular acidification and the oxygen consumption rate (ECAR & OCR), Seahorse Mito Stress Kit (Agilent) was used according to the manufacturer’s instructions. BMDMs were treated with mitochondrial complex V inhibitor oligomycin (Oligio), mitochondrial membrane uncoupling agent carbonyl cyanide p-trifluoromethoxyphenylhydrazone (FCCP) and finally complex I and III inhibitors rotenone and antimycin A (R + AA)^37,72^. In addition, a 1 h pre-treatment with p38 ATPase inhibitor SB202190 (50 μM) prior to incubation with VWF or LPS was performed where indicated. Mitochondrial morphology was determined using a Leica SP8 scanning confocal microscopy under live cell imaging. BMDM were seeded onto 4 well culture dishes (Ibidi) at 1 × 10^5^ cells /well. Cells were stained with MitoTracker™ Red (Molecular Probes, Thermo fisher) and 20 images were taken per treatment. Images were analyzed for mitochondrial morphology using Fiji ImageJ software. On average 60 mitochondrias were measured per cell. Mitochondrial fragmentation was considered <1 μm, and elongated >3 μm^39,40^.

### Role of VWF administration on peritoneal immune cell recruitment

All animal experiments were approved by the Animal Research Ethics Committee, Trinity College Dublin, and were performed in compliance with the Health Product Regulatory Authority, Ireland. In brief, adult 8–12 weeks old female C57BL/6J strain mice were used in all experiments. Animals were housed in a specific pathogen-free facility in individually ventilated and filtered cages under positive pressure. Mice were injected intraperitoneally with clinical-grade rVWF (2 mg/kg) or PBS. After 3 and 24 h, mice were culled, and lavage solution (PBS) was injected into the peritoneum and cells recovered. The lavage fluid containing peritoneal exudate cells (PECs) was centrifuged at 400 × *g* for 10 min at 4 °C, and cells were counted and processed for flow cytometry and qPCR.

Surface marker expression on peritoneal cells was assessed by flow cytometry (Supplementary Fig. 10)^73,74^. PECs were resuspended in Fc-block anti-mouse CD16/CD32 mAb (Invitrogen, Waltham, USA). Cells were stained with anti-F4/80-FITC (Biolegend, San Diego, USA), anti-Siglec F-APC (Invitrogen), anti-CD11b-APC-Cy7 (Biolegend), anti-Ly6G-BV650 (Biolegend), anti-CD45-BV711 (Biolegend), anti-MHC-II-PE (Biolegend). A live/dead marker (Life/Dead Aqua; Thermo Scientific, Waltham, USA) was included to ensure analysis of viable cells only. PEC cells were gated as eosinophils (CD45^+^CD11b^-^SiglecF^+^), neutrophils (CD45^+^CD11b^+^SiglecF^-^Ly6G^+^), macrophages (CD45^+^CD11b^+^SiglecF^-^Ly6G^-^F4/80^+^), small peritoneal macrophage (SPM; CD45^+^CD11b^+^SiglecF^-^Ly6G^-^F4/80^low^MHCII^hi^) and large peritoneal macrophage populations (LPM; CD45^+^CD11b^+^SiglecF^-^Ly6G^-^F4/80^hi^MHCII^lo^) Cells were acquired using a BD LSR Fortessa flow cytometer (BD Biosciences, Franklin Lakes, USA) followed by analysis with FlowJo software (Tree Star, OR USA).

### Data presentation and statistical analysis

All experimental data and statistical analysis were performed using the GraphPad Prism program (Graphpad Prism version 5.0 for Windows; GraphPad Software, Inc. San Diego, CA). Data were expressed as mean values ± standard deviation (SD). To assess statistical differences, data were analyzed using Student’s unpaired two-tailed *t* test or by ANOVA to compare means of three or more groups following Kolmogorov-Smirnov or Shapiro-Wilk tests for normality. For all statistical tests, *P* values < 0.05 were considered significant.

### Reporting summary

Further information on research design is available in the Nature Research Reporting Summary linked to this article.

## Supplementary information

Supplementary Information

Reporting Summary

## Data Availability

The data generated in this study are provided in the Supplementary Information/Source Data file. The RNA-Seq data files have been submitted to NCBI’s Gene Expression Omnibus and are stored under accession number GSE205365.

## Source data

Source data
