# Supplementary material for: von Willebrand factor links primary hemostasis to innate immunity
Source: Nat Commun. 2022 Nov 3;13:6320. doi: 10.1038/s41467-022-33796-7 (PMC9633696; doi:10.1038/s41467-022-33796-7)
Supplement: Supplementary file 1 — Supplementary Information [file 41467_2022_33796_MOESM1_ESM.pdf]

VON WILLEBRAND FACTOR LINKS PRIMARY HEMOSTASIS TO INNATE IMMUNITY

Clive Drakeford[1]*, Sonia Aguila[1,2]*, Fiona Roche,[3] Karsten Hokamp,[3] Judicael Fazavana,[1] Mariana P. Cervantes[4], Annie M. Curtis[4], Heike C. Hawerkamp[5], Sukhraj Pal Singh Dhami,[1] Hugo Charles-Messance[6], Emer E. Hackett[6], Alain Chion[1], Soracha Ward[1], Azaz Ahmad[1], Ingmar Schoen[1], Eamon Breen[7], Joe Keane[5], Ross Murphy[8], Roger J.S. Preston[1], Jamie M. O'Sullivan[1], Frederick J. Sheedy[6], Padraic Fallon[5,8] and James S. O'Donnell[1,9,10]

[1] Irish Centre for Vascular Biology, School of Pharmacy and Biomolecular Science (PBS), Royal College of Surgeons in Ireland.

[2] Centro Regional de Hemodonación, Hospital Universitario Morales Meseguer, IMIB-Arrixaca, Murcia, Spain.

[3] Smurfit Institute of Genetics, School of Genetics and Microbiology, Trinity College Dublin, College Green, Dublin, Ireland.

[4] School of Pharmacy and Biomolecular Science (PBS) and Tissue Engineering Research Group (TERG), Royal College of Surgeons in Ireland, Dublin 2, Ireland.

[5] School of Medicine, Trinity College Dublin, Dublin 2, Ireland.

[6] School of Biochemistry and Immunology, Trinity Biomedical Sciences Institute, Trinity College Dublin, Ireland.

[7] Department of Clinical Medicine, Trinity Translational Medicine Institute, Trinity College Dublin, Dublin, Ireland.

[8] Department of Cardiology, St James's Hospital, Dublin, Ireland.

[9] National Children's Research Centre, Our Lady's Children's Hospital, Dublin, Ireland.

[10] National Coagulation Centre, St James's Hospital, Dublin, Ireland.

SUPPLEMENTARY MATERIALS

Supplementary Figure 1

a

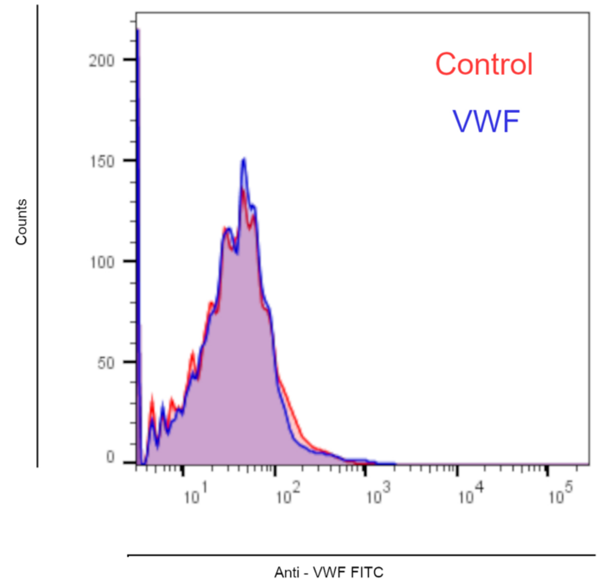

b

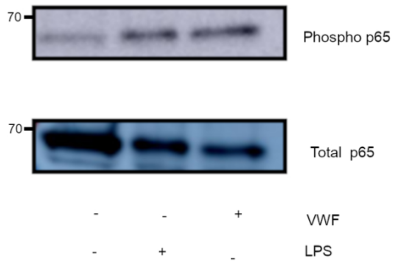

Supplementary Figure 1: Human VWF does not bind to undifferentiated primary human monocytes but induces p65 phosphorylation in primary human macrophages

(A) Binding of plasma-derived VWF (pd-VWF) to undifferentiated primary monocytes from human was assessed in vitro using by flow cytometry. A representative histogram is presented where red represents control cells not treated with VWF and blue cells treated with VWF. In contrast to macrophages, no VWF binding to primary monocytes was observed.

(B) Primary human macrophages were incubated with VWF (10µg/ml for 30mins) and then p65 phosphorylation and total p65 were assessed by Western blotting. All experiments were performed in triplicate and source data for this figure are provided as a Source Data file.

Supplementary Figure 2

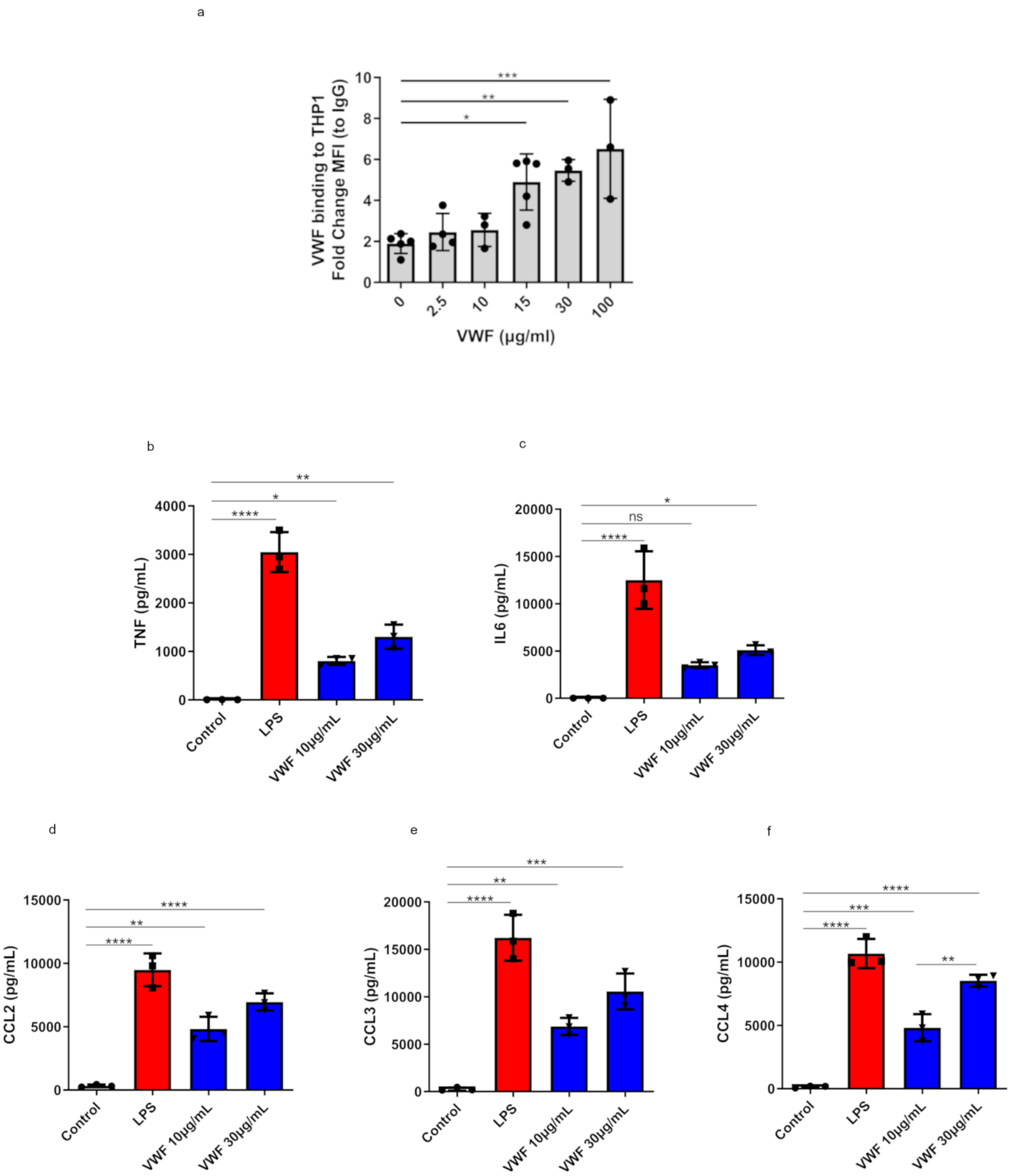

Supplementary Figure 2: Dose dependent binding and pro-inflammatory effects of VWF in macrophages

(A) Dose-dependent binding of VWF to THP-1 macrophages. (B-E) BMDM were incubated with pd-VWF (10-30 μg/ml) or LPS (100ng/ml) for 24 hours and secretion of TNF (*P=0.0167, ***P=0.0008 for 10 and 30μg/ml VWF, respectively), IL-6 (P=0.0934, *P=0.0164 for 10 and 30μg/ml VWF, respectively), CCL2 (**P=0.0011, ****P<0.0001 for 10 and 30μg/ml VWF, respectively), CCL3 (**P=0.043, ***P=0.0002 for 10 and 30μg/ml VWF, respectively) and CCL4 (***P=0.0005, ****P<0.0001 for 10 and 30μg/ml VWF, respectively) assessed using commercial ELISA assays. All experiments were performed in triplicate, and the results showed represent the mean values ± SD; *P < 0.05, **P < 0.01, ***P < 0.001, ****P<0.0001 respectively. Source data for this figure are provided as a Source Data file.

Supplementary Figure 3

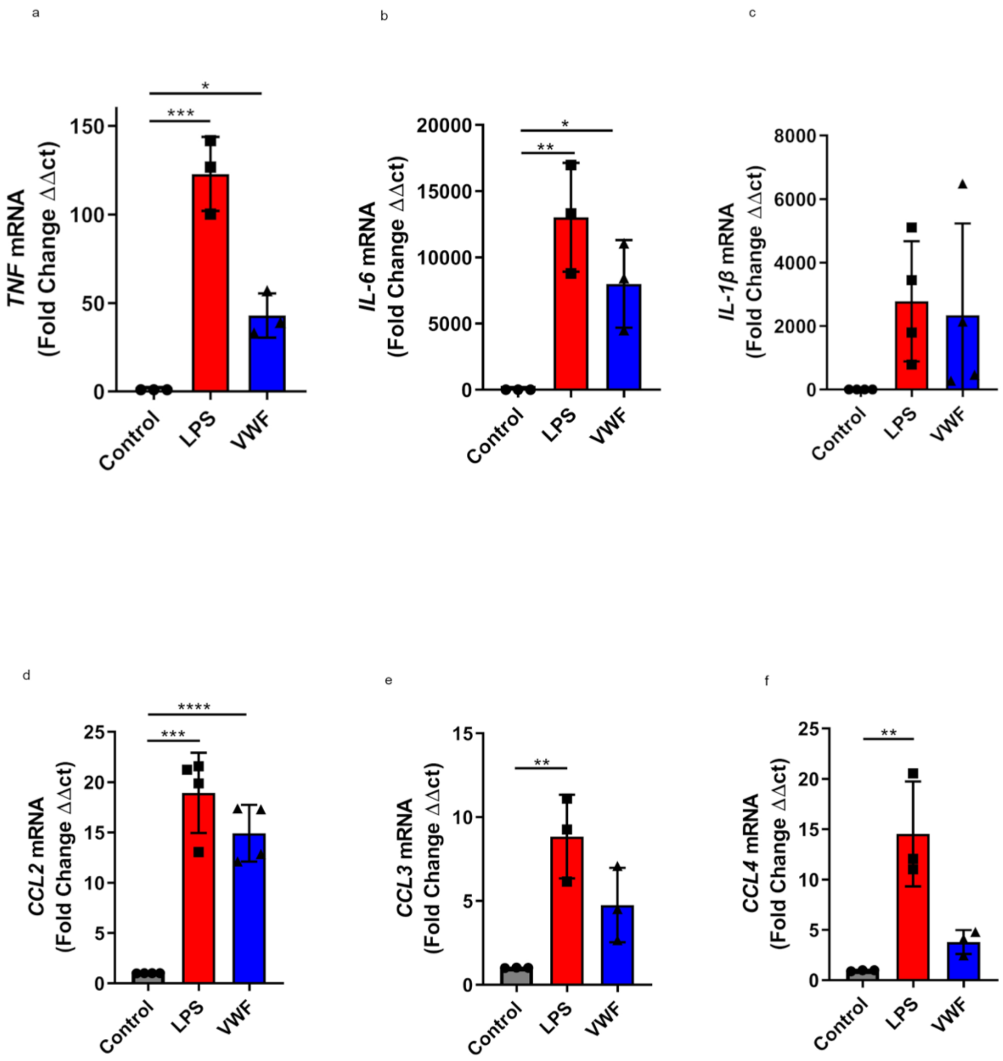

Supplementary Figure 3: VWF induces pro-inflammatory cytokine and chemokine expression in macrophages.
(A) pd-VWF (10µg/ml) or LPS (100ng/ml) incubation for 4 or 24 hours with primary human macrophages significantly induced *TNF* expression compared to control (*P*=0.025 and *P*=0.0001, respectively). **B)** Furthermore, pd-VWF significantly enhanced *IL-6* expression (*P*=0.041 and *P*=0.0046, respectively) to similar levels as LPS induction. **(C)** Incubation with both VWF and LPS was associated with non-significant increases in macrophage *IL-1β* mRNA (*P*=0.27 and *P*=0.17, respectively). Similarly, VWF treatment was also associated with increase in expression levels chemokine, CCL2. **(D)** *CCL2* (*P*=0.0002 and *P*<0.0001, respectively), **(E)** *CCL3* (*P*=0.11 and *P*=0.006, respectively) and **(F)** *CCL4* (*P*=0.53 and *P*=0.004, respectively). All experiments were performed in triplicate, and the results showed represent the mean values ± SD. **P* < 0.05, ***P* < 0.01, ****P* < 0.001, *****P* < 0.0001 respectively. Source data for this figure are provided as a Source Data file.

Supplementary Figure 4

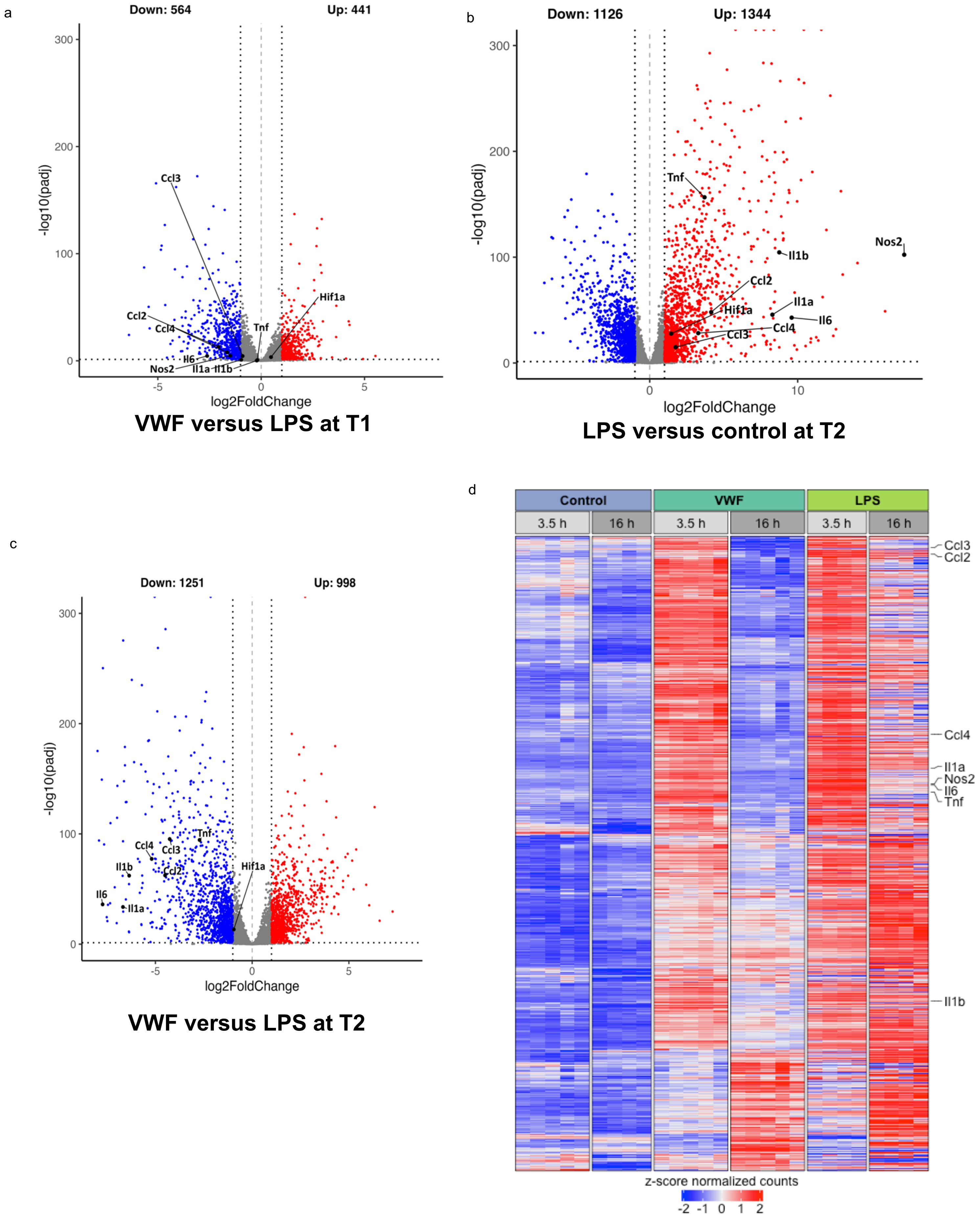

Supplementary Figure 4: VWF induces a transcriptional response in macrophages that is distinct from the LPS response.
Volcano plots of significantly differentially expressed genes (FDR < 0.05) as detected by RNAseq in (A) VWF-treated macrophages versus LPS for 3.5 hrs, (B) LPS-treated macrophages versus PBS control for 16 hrs, and (C) VWF-treated macrophages versus LPS for 16 hrs. Red and blue dots indicate statistically significant up- and down-regulated genes, respectively. Key pro-inflammatory genes are labelled in black. (D) Heatmap of genes that are induced by VWF-treated macrophages versus control. Gene order is based on heatmap in Figure 2C. Key proinflammatory genes are listed along the heatmap. Expression values are represented as the z-score of normalized counts. Source data for this figure are provided as a Source Data file.

Supplementary Figure 5

a

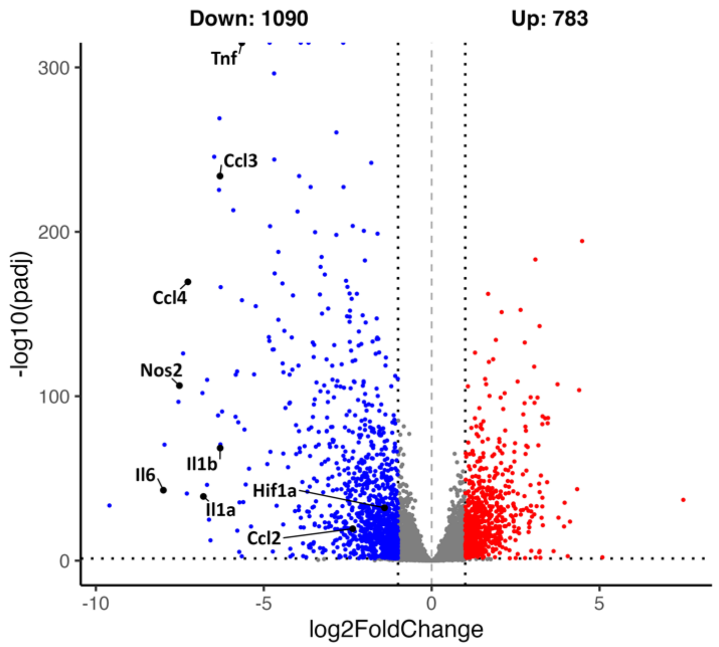

VWF T2 versus VWF T1

b

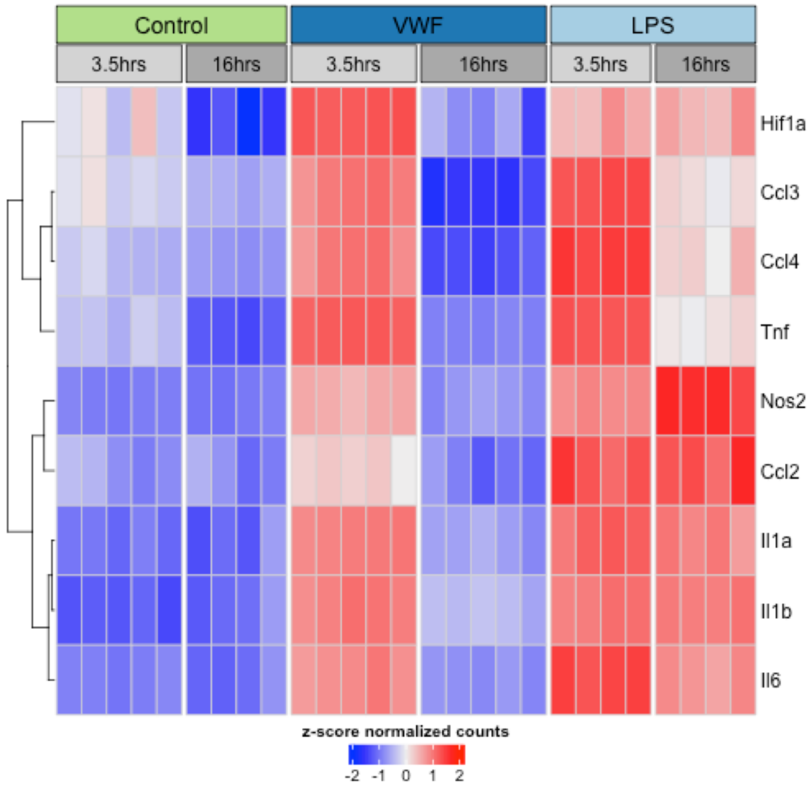

Supplementary Figure 5: VWF induces an early proinflammatory signal in macrophages that is switched off by 16 hrs.

(A) Volcano plot of significantly differentially expressed genes (FDR < 0.05) as detected by RNAseq in VWF-treated macrophages at 16 hrs versus 3.5 hrs. All key proinflammatory genes are downregulated at 16 hrs compared to 3.5 hrs. **(B)** Heatmap of key proinflammatory genes in PBS control-treated, VWF-treated and LPS-treated macrophages at different time points. Expression values are represented as the z-score of normalized counts. Source data for this figure are provided as a Source Data file.

Supplementary Figure 6

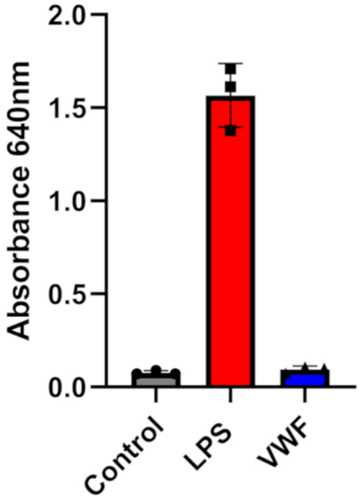

Supplementary Figure 6: Commercial purified pd-VWF is endotoxin free.
Commercial purified pd-VWF was tested for contaminating endotoxin using macrophage a RAW-Blue cell line (InvivoGen). RAW-Blue cells were incubated with pd-VWF (10µg/ml) or LPS (100ng/ml) overnight. Secreted embryonic alkaline phosphatase (SEAP) was quantified using QUANTI-Blue (InvivoGen). Experiments were performed in triplicate, and the results showed represent the mean values ± standard deviation (SD).Source data for this figure are provided as a Source Data file.

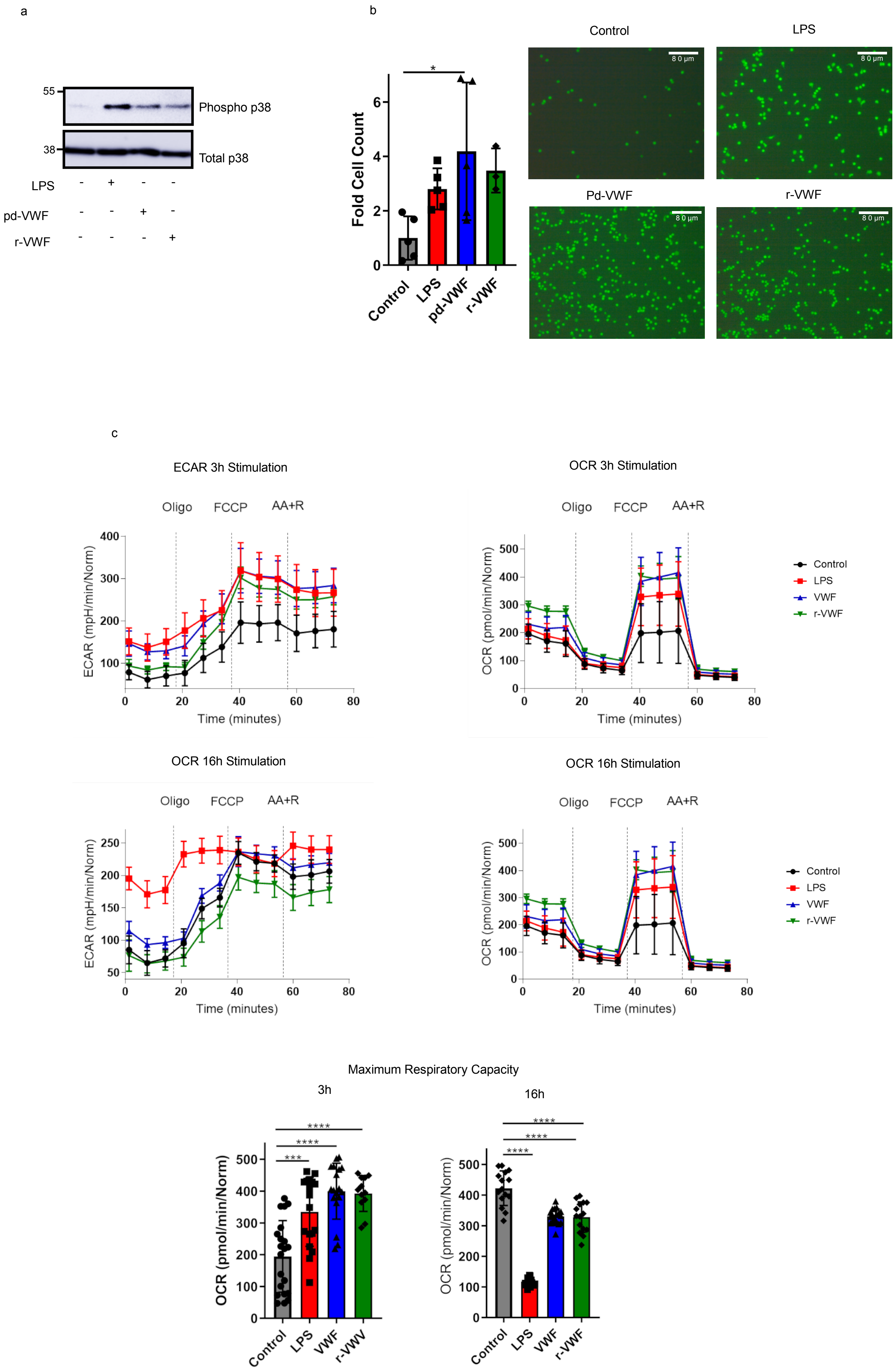
a
55
38
Phospho p38
Total p38
LPS - + - -
pd-VWF - - + -
r-VWF - - - +
b
Fold Cell Count
*
Control
LPS
pd-VWF
r-VWF
Control
LPS
Pd-VWF
r-VWF
80 µm
c
ECAR 3h Stimulation
OCR 3h Stimulation
Oligo
FCCP
AA+R
ECAR (mpH/min/Norm)
OCR (pmol/min/Norm)
Time (minutes)
Control
LPS
VWF
r-VWF
OCR 16h Stimulation
OCR 16h Stimulation
Maximum Respiratory Capacity
3h
16h
OCR (pmol/min/Norm)
***
****
Control
LPS
VWF
r-VWV
r-VWF

Supplementary Figure 7: Binding of clinical-grade recombinant VWF to macrophages was associated with pro-inflammatory changes.

(A) Western blot analysis of phosphorylation of MAPKinase p38 in primary human macrophages incubated with recombinant clinical grade, r-VWF (10 µg/ml) compared to either pd-VWF (10 µg/ml) or LPS (100 ng/ml) for 30min. (B) Primary human macrophages were incubated with recombinant VWF (10 µg/ml), pd-VWF (10 µg/ml) or LPS (100 ng/ml) for 24 hours. Cell supernatants were then collected and placed in the lower chamber of a transmigration wells. Naïve human monocytes from human blood were placed in the upper chamber and allowed to migrate for 2.5 hours. Representative images of migrated cell tracker green ladled primary monocytes. Bar charts are a pool of 3-5 independent experiments (mean ± SD) relativized to control. (C) Extracellular flux analysis (Seahorse XF, Cell Mito Stress kit) was used to assess the effects of recombinant VWF (r-VWF) binding upon macrophage metabolism. Extracellular acidification rate (ECAR) measured to study the effects on glycolysis following stimulation with r-VWF (10µg/ml) (green), pd-VWF (10µg/ml) (blue), LPS (100ng/ml) (red), or untreated controls (black) for 3 hours and 16 hours respectively. Similarly, cellular oxygen consumption rate (OCR) was assayed to study the effects on oxidative phosphorylation after 3 hours and 16 hours incubations. The effects of different VWF and LPS on BMDMs glycolysis and oxidative phosphorylation were studied in the presence or absence of specific mitochondrial inhibitors as before. Plots are representative images collected from two or three independent assays. The significance for all assays was determined by ANOVA in which *p<0.05, **p<0.01, ***p<0.001, ****p<0.0001. All scale bars are 80µm. Source data for this figure are provided as a Source Data file.

Supplementary Figure 8

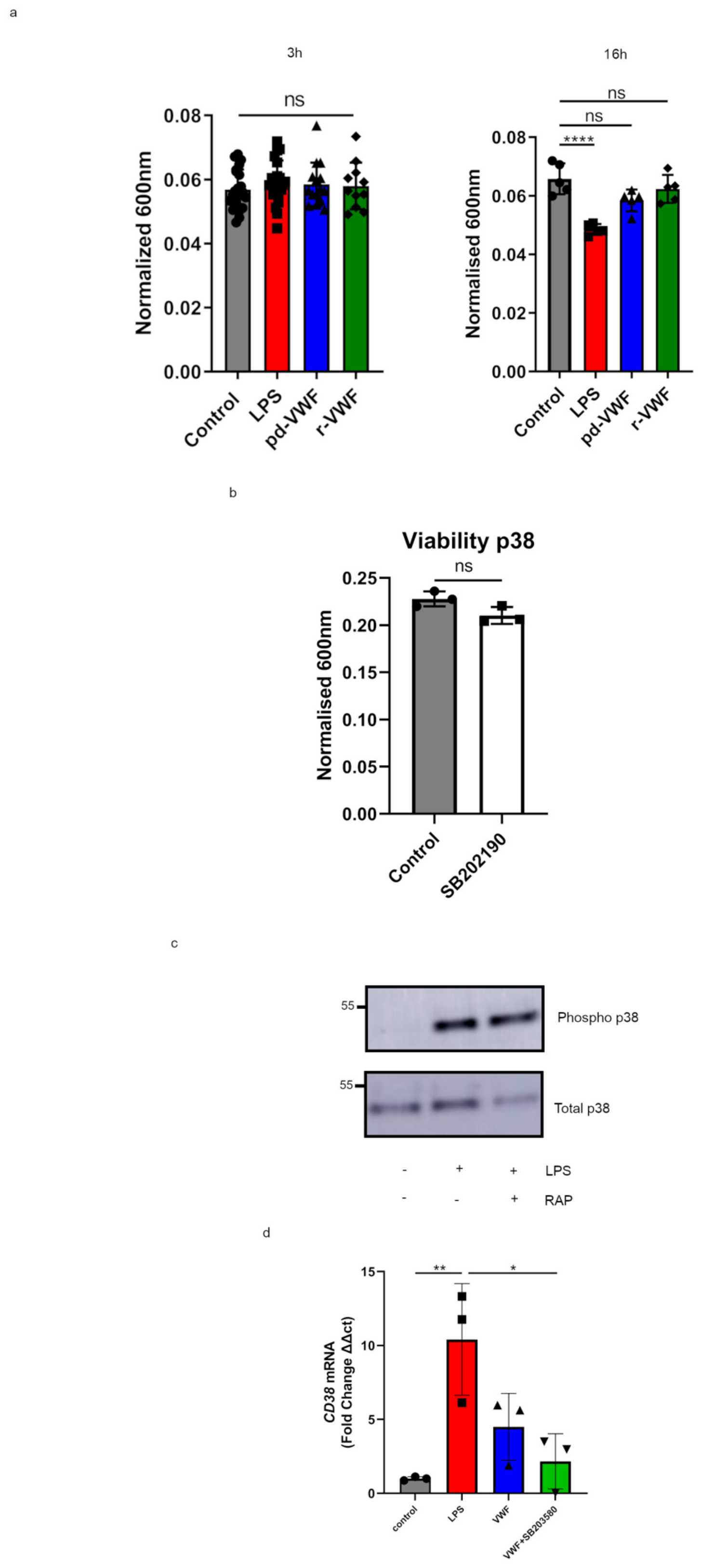

Supplementary Figure 8: VWF does not affect macrophage viability but promotes pro-inflammatory signalling through the LRP1 receptor.
(A) BMDM viability was assessed after incubations with r-VWF (10µg/ml) (green), pd-VWF (10µg/ml) (blue), LPS (100ng/ml) (red), or untreated controls (dark grey) for 3 hours and 16 hours. (B) BMDM viability was also determined following 3 hours treatment with p38 inhibitor (SB202190). Cell viability was determined using Alamar Blue metabolism (absorbance λ = 600 nm) normalised to total protein. (C) To examine whether LPS requires LRP1 for p38 activation in macrophages, BMDM were treated with LPS (100ng/ml for 30mins) in the presence or absence of RAP (200nM) for 30 mins. Cells were lysed and blotted for phopho-p38 and total p38 respectively. (D) To investigate whether p38 signalling is involved in mediating the M1 phenotype induced by VWF treatment, human macrophages were treated with pd-VWF (10µg/ml) in the presence or absence of p38 antagonist SB203580 (50uM) for 3 hours and CD38 mRNA quantified qPCR. The significance of different assays was calculated by ANOVA or t-test, where ****P<0.0001 (ns=not significant). Source data for this figure are provided as a Source Data file.

Supplementary Figure 9

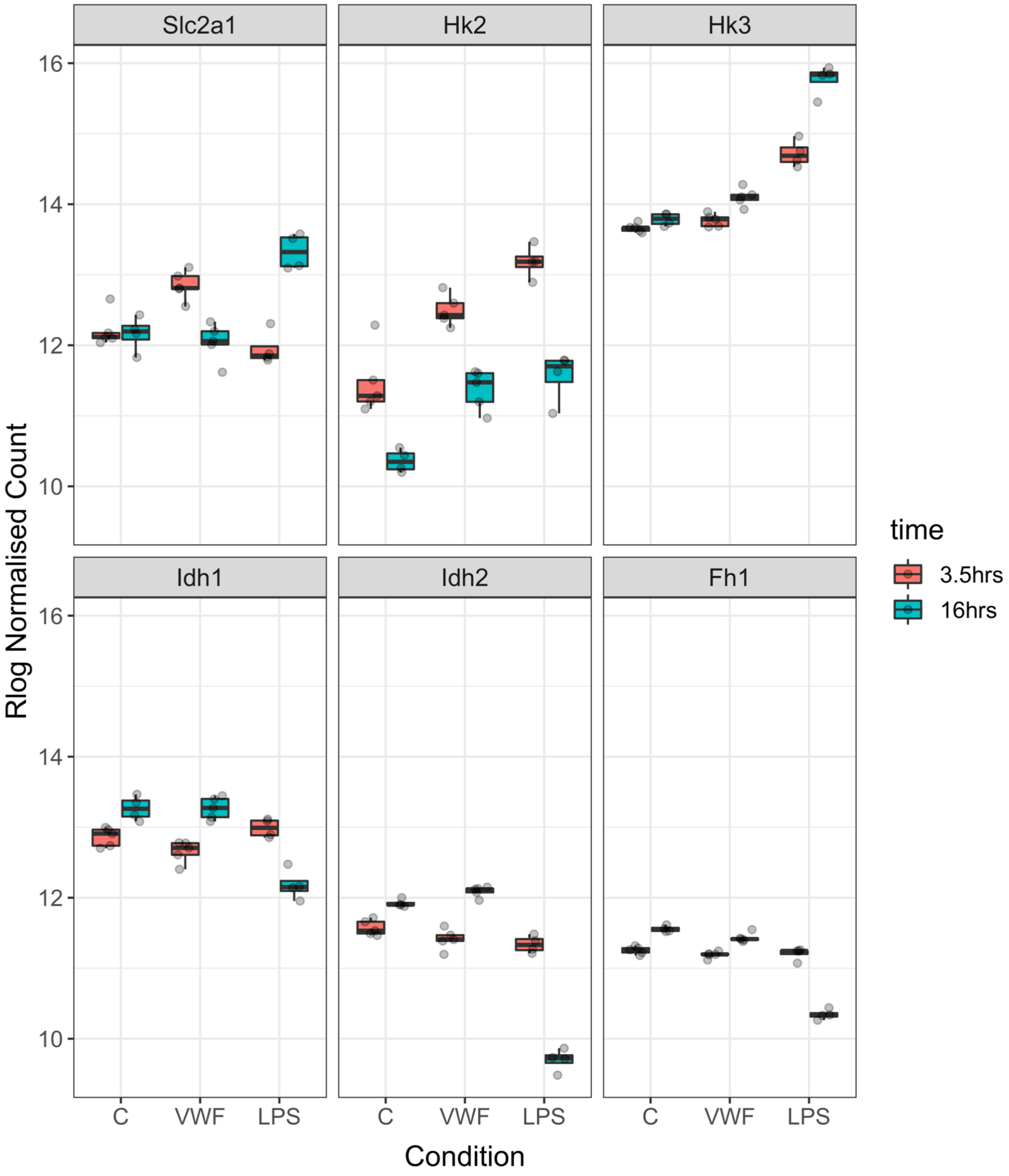

Expression changes on key metabolic genes associated with glycolysis and TCA cycle.
Boxplot of rlog normalized read counts of key glycolytic genes (Slc2a1, Hk2, Hk3) and TCA cycle genes (Idh1, Idh2, Fh1) from control, VWF and LPS-treated macrophages across different time points.

Supplementary Figure 10

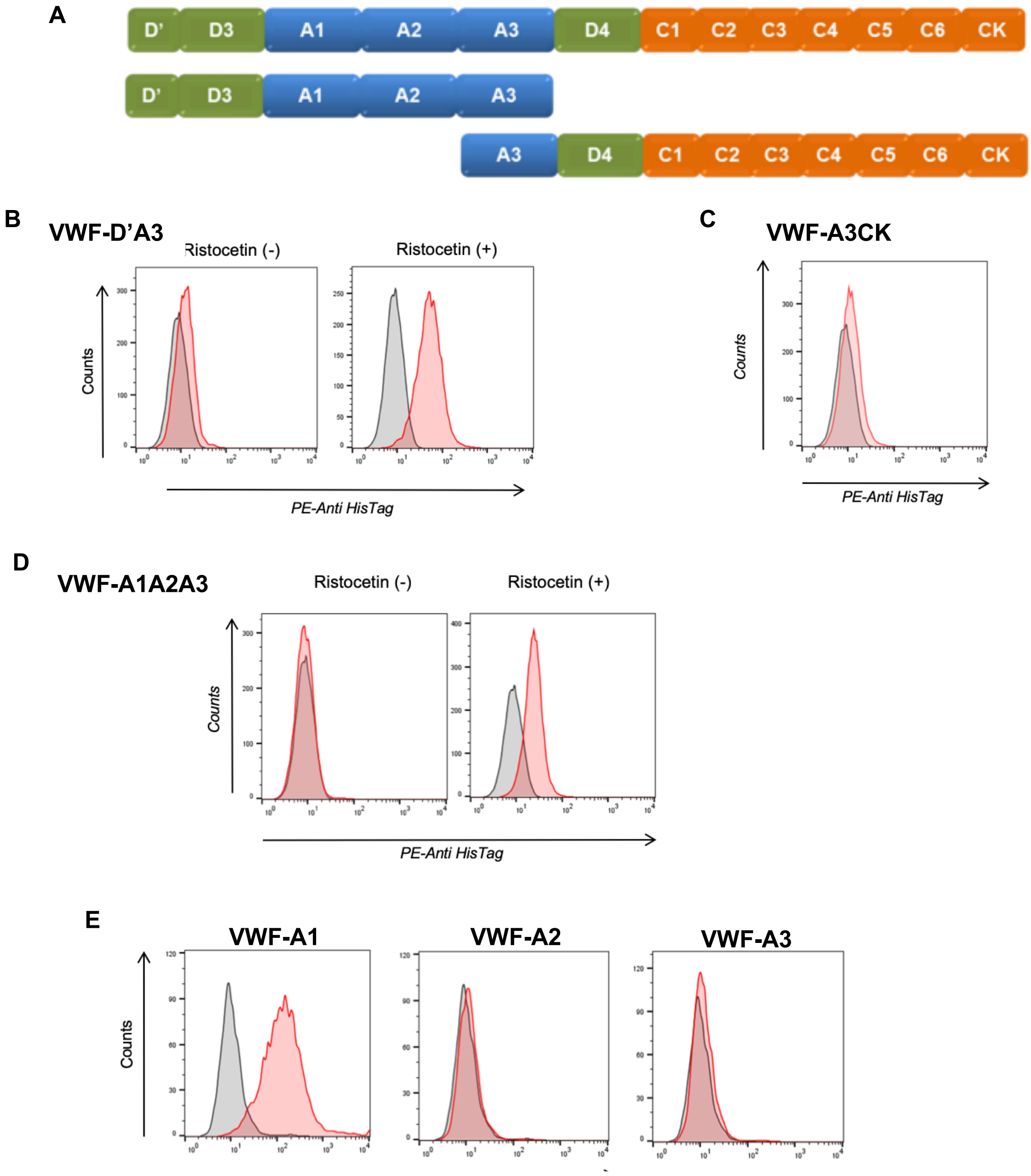

Supplementary Figure 10: Multiple VWF domains contribute to macrophage binding. (A) Each VWF monomer is composed of a series of individual domains. (**B-E**) To examine the role of VWF domains in modulating interaction with macrophages, a series of selected recombinant VWF truncations were expressed were transiently expressed in HEK293T cells. Conditioned serum free medium was harvested, concentrated and further purified via nickel affinity chromatography. Subsequently, binding of VWF truncations to primary macrophages was assessed using flow cytometry. Where indicated binding studies were performed in the presence or absence of ristocetin (1.5mg/ml).

Supplementary Figure 11

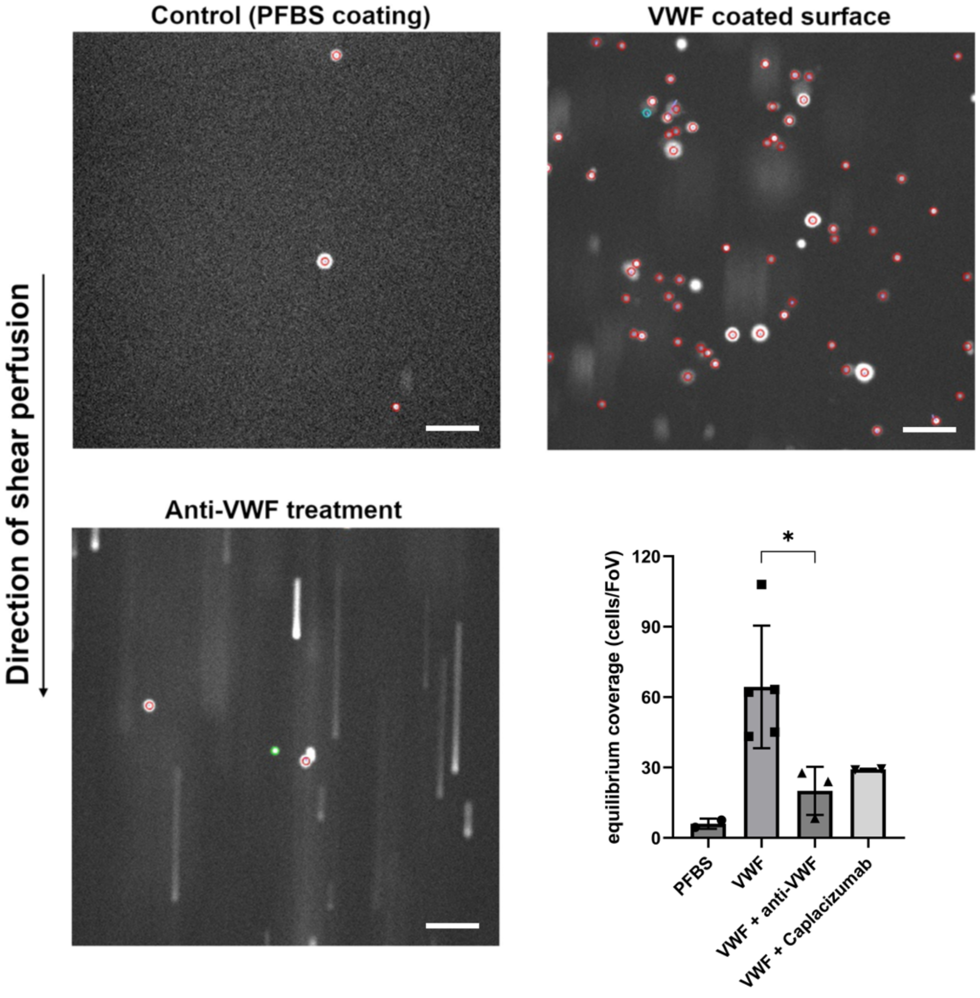

Supplementary Figure 11: Multiple VWF domains are involved in mediating macrophage binding under shear stress.
To examine VWF interaction with macrophages under shear, THP-1 cells were resuspended in differentiation media (serum-free RPMI media, 0.1% BSA, 1mM $MnCl_2$ and 100nM PMA) for 15 minutes at 37°C. Differentiated THP-1 cells were then fluorescently labelled. pd-VWF in Protein Free Blocking solution (PFBS) was coated onto µ-slides VI 0.4 and differentiated THP-1 cells perfused across the channels at 0.25ml/min. VWF-adhered THP-1 cells were observed in real-time using an EM-CCD camera (Andor iXon 888) on Zeiss Axiovert microscope system with Metamorph v7.8.2 software. Time-lapse image sequences were acquired at 32 frames per second using a 10x Plan Neofluar (NA 0.3) objective. Where indicated, shear perfusion experiments were performed in the presence of polyclonal anti-VWF (P=0.0336 compared to VWF alone) or anti-A1 domain antibodies (20µg/ml). All scale bars are 100 µm. Source data for this figure are provided as a Source Data file.

Supplementary Figure 12

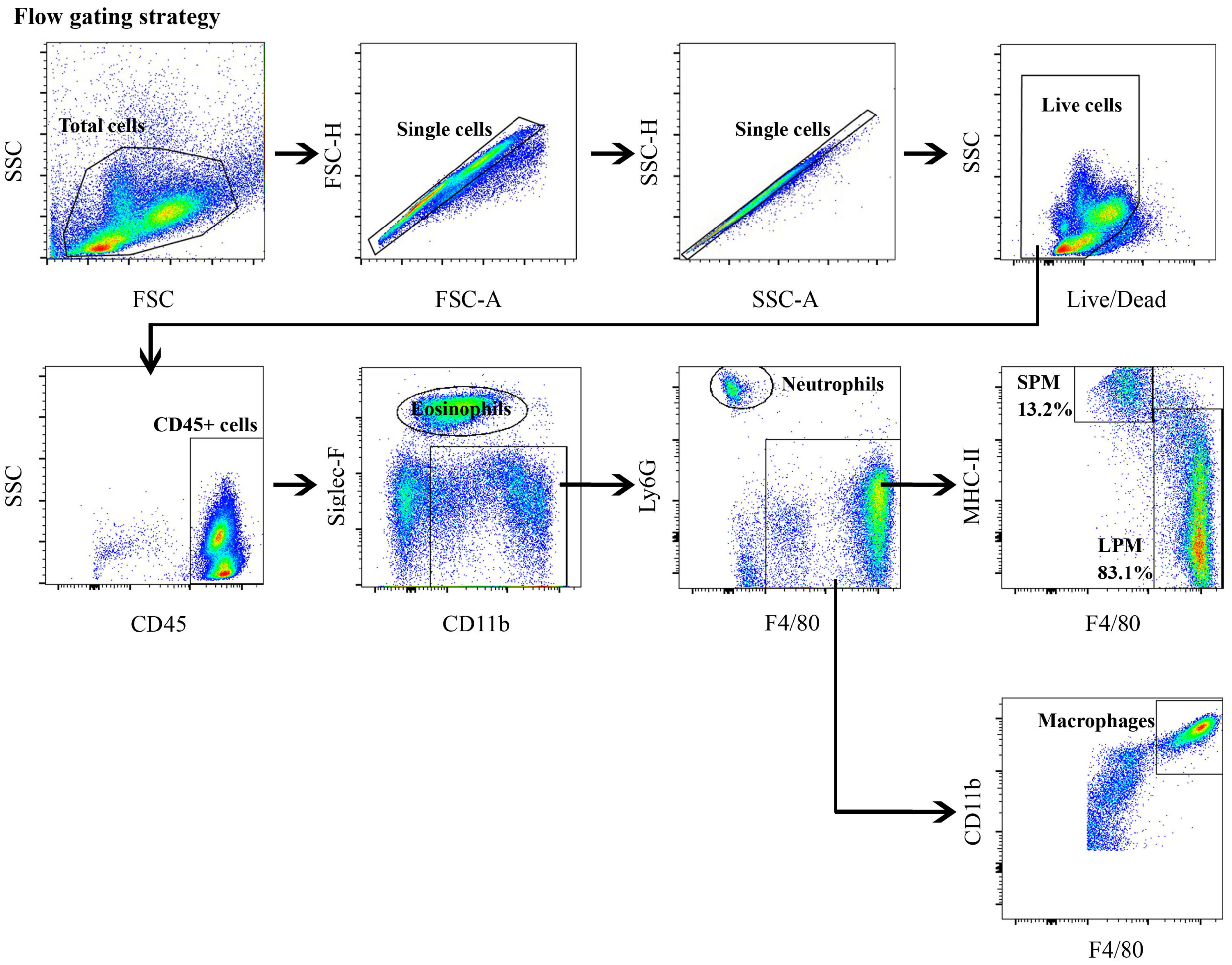

Supplementary Figure 12: Flow cytometry gating strategy for cells isolated from the peritoneum of mice.

Flow cytometry gating strategy for cells isolated from the peritoneal of mice. In brief, peritoneal exudate cells were collected by lavage in sterile PBS. Initial gating strategy to remove cell doublets and dead cells and CD45+ cells were defined as eosinophils ($CD11b^{-}SiglecF^{hi}$), neutrophils ($CD11b^{+}SiglecF^{-}F4/80^{-}Ly6G^{+}$), macrophages ($CD11b^{+}SiglecF^{-}Ly6G^{-}F4/80^{+}$) with of identification of frequency of large peritoneal macrophages (LPM; $CD11b^{+}SiglecF^{-}Ly6G^{-}F4/80^{hi}MHCII^{lo}$) and small peritoneal macrophages (SPM; $CD11b^{+}SiglecF^{-}Ly6G^{-}F4/80^{low}MHCII^{hi}$.

Supplementary Figure 13

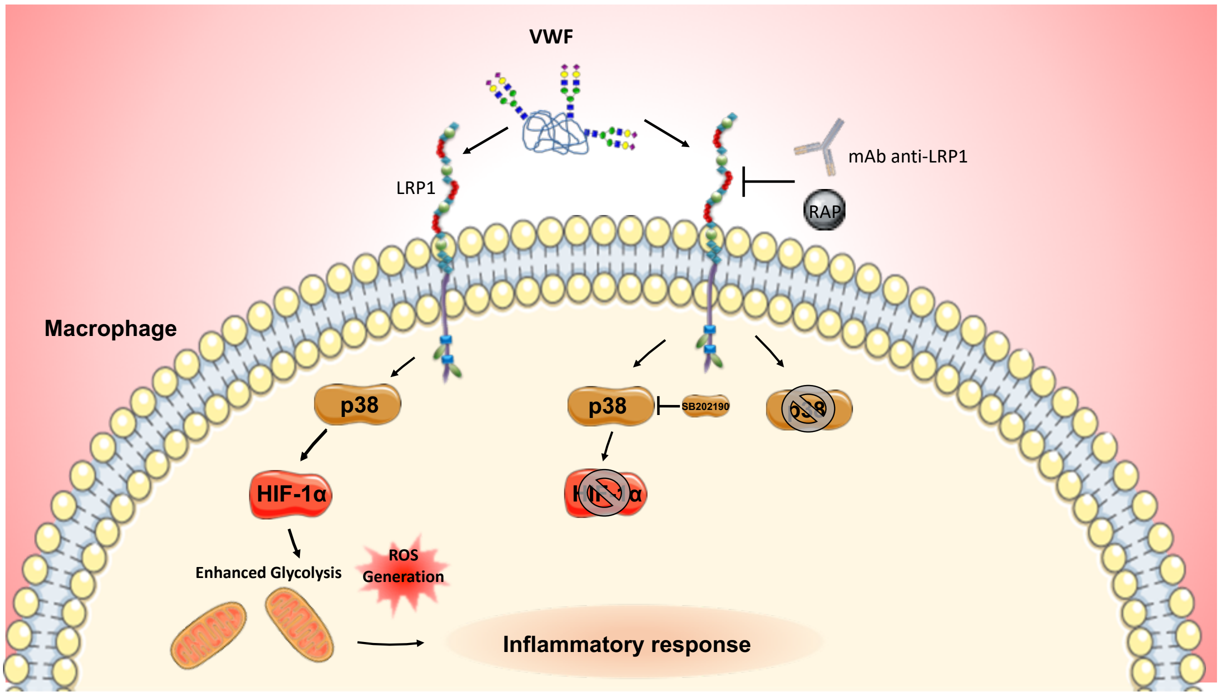

Supplementary Figure 13 Graphical abstract

Following vascular injury, the plasma sialoglycoprotein VWF comes into contact with tissue macrophages to which it can bind via a variety of scavenger (eg. LRP1 and SR-A1) and/or lectin (eg. MGL and Siglec-5) cell surface receptors. In particular, VWF binding to macrophages triggers pro-inflammatory signalling including the activation of the MAPKinase pathway (with phosphorylation of p38 and JNK) and NF-κB. Importantly, we have established a novel link between VWF LRP1 interaction and p38 signalling. Crucially, VWF dependent activation of p38 results in a rapid enhancement of glycolysis, mitochondrial fragmentation and ROS generation though HIF-1α accumulation. This interaction results in the adoption of a M1 phenotype, leading to enhanced expression of pro-inflammatory cytokines (including TNF and IL-6) and chemokines (including CCL2, CCL3 and CCL4). Thus, VWF not only plays a key role in initiation of primary haemostasis sites of vascular injury, but also serves as a damage signal to prime local tissue macrophages to initiate pro-inflammatory responses

Supplementary Figure 14

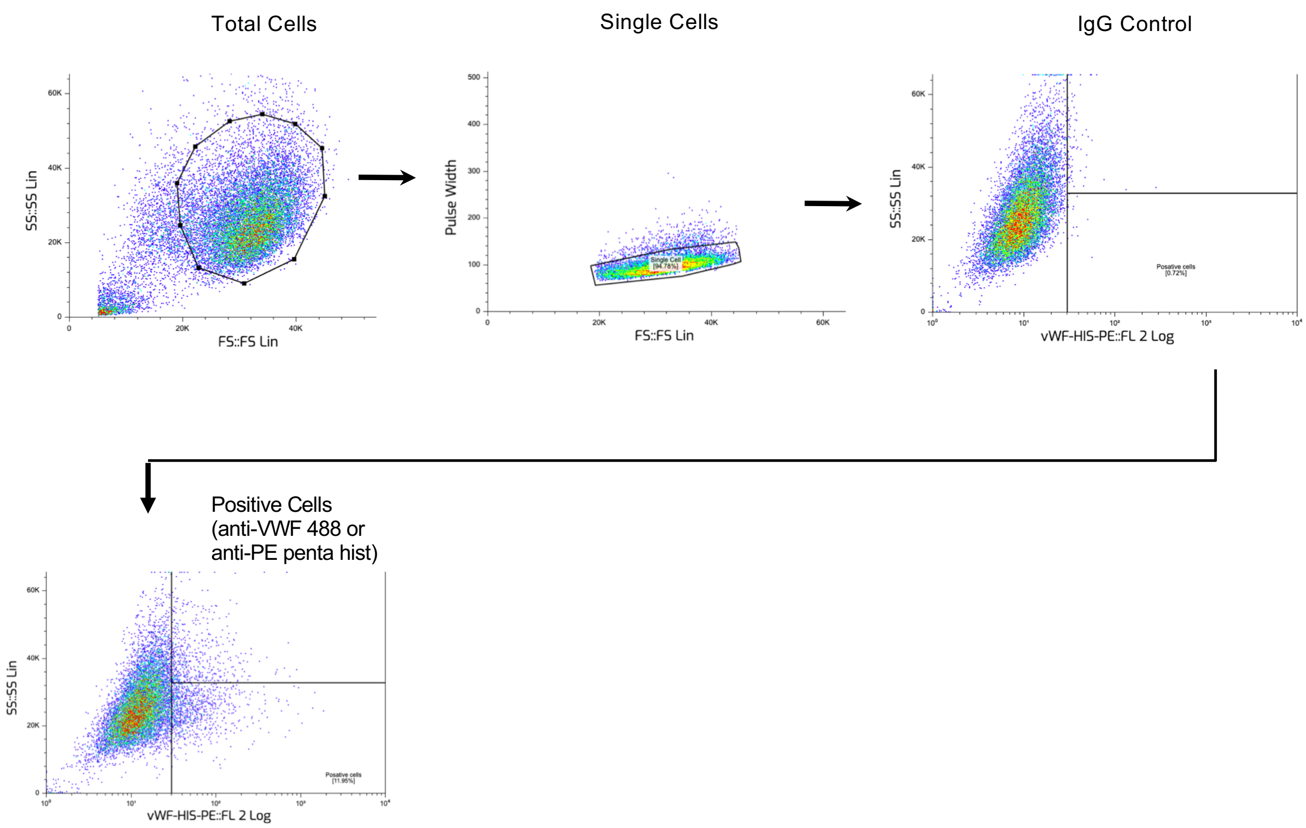

Supplementary Figure 14: Flow cytometry gating strategy for monocytes binding VWF

Supplementary Figure 15

Flow cytometry gating strategy for M1 M2

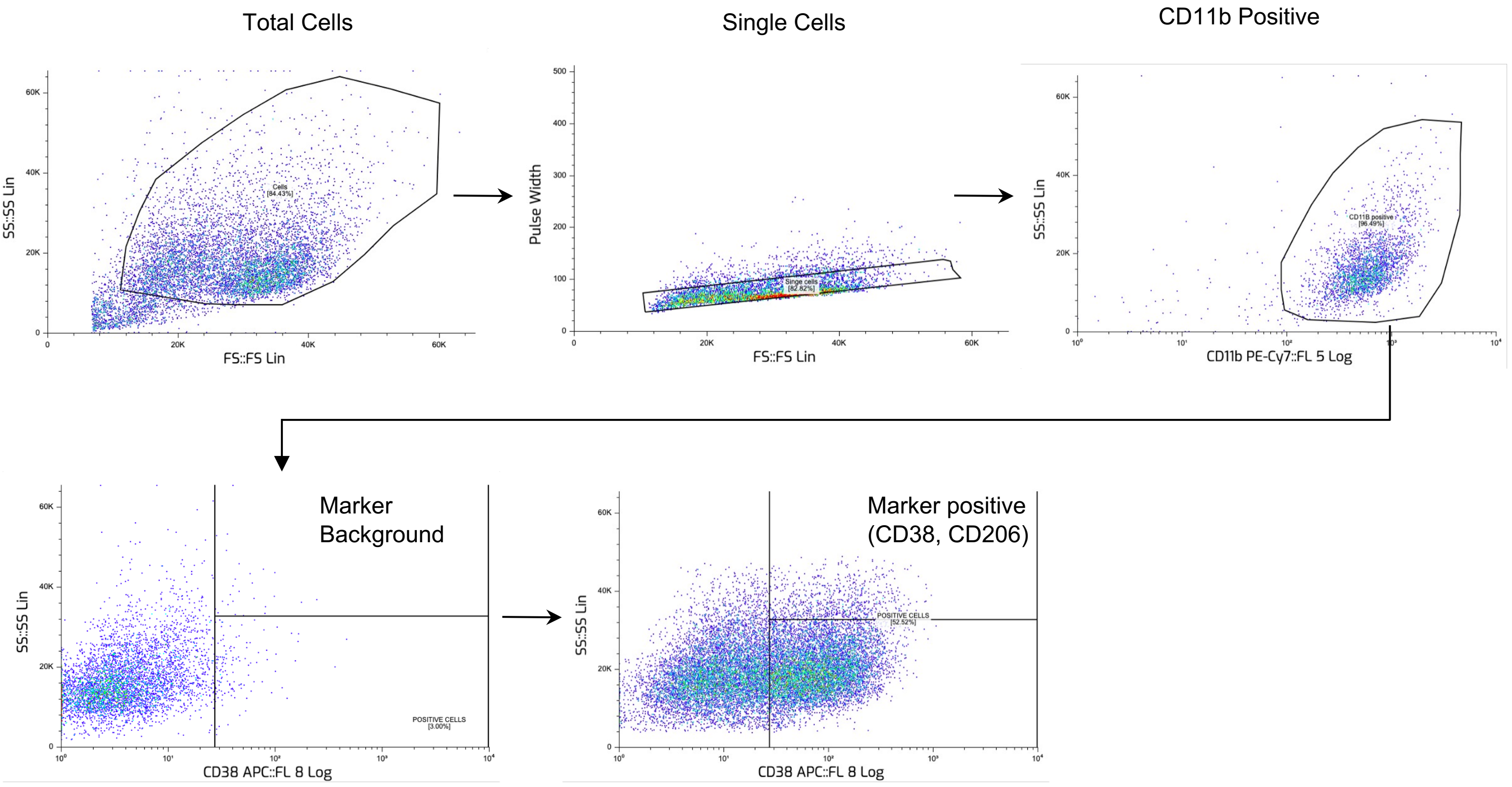

Flow cytometry gating strategy for ROS

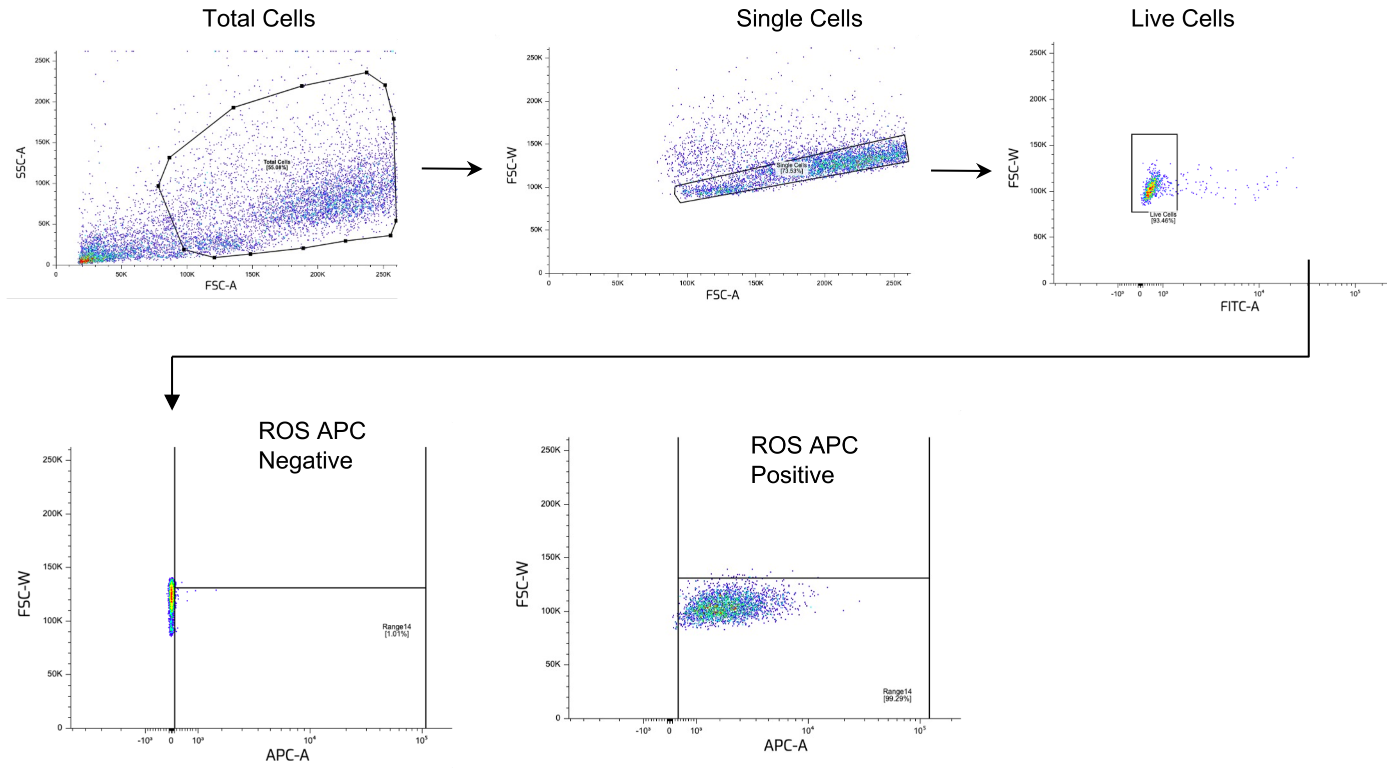

Supplementary Table 1.

Human

Gene	Forward Primer	Reverse Primer
IL-1β	CTAAACAGATGAAGTGCTCC	GGTCATTCTCCTGGAAGG
IL-6	GCAGAAAAAGGCAAAGAATC	CTACATTTGCCGAAGAGC
TNFα	AGGCAGTCAGATCATCTTC	TTATCTCTCAGCTCCAGC
CCL2	AGACTAACCCAGAAACATCC	ATTGATTGCATCTGGCTG
CCL3	GCAACCAGTTCTCTGCATCA	TGGCTGCTCGTCTCAAAGTA
CCL4	GCTTTTCTTACACTGCGAGGA	CCAGGATTCACTGGGATCAG
β-Actin	GACGACATGGAGAAAATCTG	ATGATCTGGGTCATCTTCTC
NOS2	GCTCTACACCTCCAATGTGACC	CTGCCGAGATTTGAGCCTCATG

Murine

Gene	Forward Primer	Reverse Primer
β-actin	TGCTGTCCCTGTATGCCTCT	TTGATGTCACGCACGATTTC
TNFα	ACGTCGTAGCAAACCACCAA	GAGAACCTGGGAGTAGACAAGG
IL-6	ATGAAGTTCCTCTCTGCAAGAGACT	CACTAGGTTTGCCGAGTAGATCTC
PHD3	CAACTTCCTCCTGTCCCTCA	CCTGGATAGCAAGCCACCA

Supplementary Table 2

Antibody	Company and Catalogue number	Dilution
Rabbit monoclonal anti phospho- p38 Thr180/Tyr182 (Clone D3F9)	CST, 4511	1:1000
Rabbit polyclonal anti p38 p38α, -β or -γ MAPK	CST, 9212	1:1000
Rabbit monoclonal anti phospho- NF-κB p65, Ser536 (Clone 93H1)	CST, 3033	1:1000
Rabbit monoclonal anti NF-κB p65 Glu498 (Clone D14E12)	CST, 8242)	1:1000
Mouse monoclonal anti- κBα Amino-terminal Antigen (Clone L35A5)	CST, 4814)	1:1000
Rabbit monoclonal anti phospho- IκBα Ser32 (Clone 5A5)	CST, 9246)	1:1000
Rabbit polyclonal anti anti SAPK/JNK	CST, 9252	1:1000
Rabbit polyclonal anti phospho-JNK Thr183/Tyr185	CST, 9251	1:1000
Mouse monoclonal β-actin N-terminal (Clone AC-74)	Sigma-Aldrich, A2228	1:3000
Rabbit monoclonal anti-HIF-1α Leu478 (Clone D1S7W	CST, Cat: 36169	1:1000
Mouse Monoclonal anti-LAMP-1, H4A3 (Clone H4A3)	SantaCruz, sc-20011	1:50
Mouse IgG2 PE conjugated anti-pentahis Tag (Clone J095G46)	BioLegend, 362603	1:25
Mouse IgG2 PE Isotype Ctrl (Clone MOPC-173)	BioLegend, 400244	1:25
Mouse monoclonal anti LRP1 (Clone 8G1)	Abcam, ab20384	1:1000
Mouse monoclonal ant-LRP1, (Clone LRP1-11)	Sigma L2420	1:100
Rat IgG2 anti-CD11b PE clone (Clone M1/70	BioLegend, 101207	1:20
Rat IgG2 Isotype Ctrl PE (Clone RTK4530)	BioLegend, 400636	1:20
Rat IgG2 anti CD206 brilliant violet 421, C068C2	BioLegend, 141717	1:20
Rat IgG2 anti-CD38 APC, clone 90	BioLegend, 102712	1:20
Rat IgG2 Isotype Ctrl APC (Clone RTK2758)	BioLegend, 102712, 400511	1:20
Polyclonal Fc Receptor Binding Inhibitor Antibody	Thermofisher, 14-9161-73	1:20
Polyclonal Alexa 488 anti-rabbit	ThermoFisherScientific, 11008	1:1000
Polyclonal anti mouse Alexa 594	ThermoFisherScientific, A-11032)	1:300
Polyclonal anti-rabbit IgG HRP-conjugated	R&D Systems HAF008	1:1000
Polyclonal anti-mouse IgG HRP-conjugated	R&D Systems HAF007	1:1000
Polyclonal peroxidase AffiniPure rabbit anti-Goat IgG (H+L) HRP-conjugate Antibody	Jackson immunoresearch,305-035-003	1:5000
Polclonal Rabbit Polyclonal anti-VWF	Agilent DAKO GA52761-2	1:1000
Monoclonal mouse anti-EEA1 Antibody (Clone 14/EEA1	BD Transduction Laboratories, 610457	1:100
Fc-block anti-mouse CD16/CD32 mAb (Clone FRC-4G8)	Invitrogen, 10291623	1:100
Mouse IgG1 anti-mouse F4/80-FITC mAb (Clone QA17A29)	BioLegend, 157313	1:20
Mouse IgG2a anti-mouse Siglec F-APC mAb (Clone 1RNM44N)	Invitrogen, 14-1702-82	1:20
Rat IgG2b anti-mouse/human CD11b-APC-Cy7 (Clone M1/70)	BioLegend, 101225	1:20
Rat IgG2a anti-mouse Ly6G-BV650 (Clone 1A8)	BioLegend, 127625	1:20
Mouse IgG1 anti-mouse CD45-BV711 (Clone QA17A26)	BioLegend, 157602	1:20
Mouse IgG2a anti-mouse MHC-II-PE (Clone AF6-120.1)	BioLegend, 116419	1:20
Life/Dead Aqua	ThermoFisherScientific, L34957	1:1000
