# Supplementary material for: von Willebrand factor links primary hemostasis to innate immunity
Source: Nat Commun. 2022 Nov 3;13:6320. doi: 10.1038/s41467-022-33796-7 (PMC9633696; doi:10.1038/s41467-022-33796-7)
Supplement: Supplementary file 2 — Reporting Summary [file 41467_2022_33796_MOESM2_ESM.pdf]

nature portfolio

Corresponding author(s): James O'Donnell

Last updated by author(s): 19/9/22

Reporting Summary

Nature Portfolio wishes to improve the reproducibility of the work that we publish. This form provides structure for consistency and transparency in reporting. For further information on Nature Portfolio policies, see our Editorial Policies and the Editorial Policy Checklist.

Statistics

For all statistical analyses, confirm that the following items are present in the figure legend, table legend, main text, or Methods section.

n/a	Confirmed	
☐	☒	The exact sample size (n) for each experimental group/condition, given as a discrete number and unit of measurement
☐	☒	A statement on whether measurements were taken from distinct samples or whether the same sample was measured repeatedly
☐	☒	The statistical test(s) used AND whether they are one- or two-sided<br>*Only common tests should be described solely by name; describe more complex techniques in the Methods section.*
☒	☐	A description of all covariates tested
☐	☒	A description of any assumptions or corrections, such as tests of normality and adjustment for multiple comparisons
☐	☒	A full description of the statistical parameters including central tendency (e.g. means) or other basic estimates (e.g. regression coefficient) AND variation (e.g. standard deviation) or associated estimates of uncertainty (e.g. confidence intervals)
☐	☒	For null hypothesis testing, the test statistic (e.g. F, t, r) with confidence intervals, effect sizes, degrees of freedom and P value noted<br>*Give P values as exact values whenever suitable.*
☒	☐	For Bayesian analysis, information on the choice of priors and Markov chain Monte Carlo settings
☒	☐	For hierarchical and complex designs, identification of the appropriate level for tests and full reporting of outcomes
☐	☒	Estimates of effect sizes (e.g. Cohen's d, Pearson's r), indicating how they were calculated

Our web collection on statistics for biologists contains articles on many of the points above.

Software and code

Policy information about availability of computer code

Data collection	NApplied Biosystems 7500 or 7900 HT was used to collect RT-PCR data<br>XFe96 analyzer (Agilent) was used to perform metabolic assays.<br>Amersham Imager 600 was used to collect western blot images.<br>Leica SP8 scanning confocal microscopy was used to get confocal images.<br>BD Biosciences FACSCanto II cytometer or Cytek Biosciences Aurora spectral analyser was used for flow cytometry data<br>VICTOR3 Multilabel Plate Reader was used for ELISAs.
Data analysis	GraphPad PRISM 8 or 9 was used to perform statistical analysis and plot results.<br>ImageStudioLite was used to perform western blot quantifications.<br>7500 SDS v1.4.1 was used to analyze RT-PCR data.<br>FlowJo v10 software was used for FACs measurements.<br>ImageJ was used to quantify confocal data

For manuscripts utilizing custom algorithms or software that are central to the research but not yet described in published literature, software must be made available to editors and reviewers. We strongly encourage code deposition in a community repository (e.g. GitHub). See the Nature Portfolio guidelines for submitting code & software for further information.

Data

Policy information about availability of data

All manuscripts must include a data availability statement. This statement should provide the following information, where applicable:

- Accession codes, unique identifiers, or web links for publicly available datasets
- A description of any restrictions on data availability
- For clinical datasets or third party data, please ensure that the statement adheres to our policy

The RNA-Seq data files have been submitted to NCBI's Gene Expression Omnibus and are stored under accession number GSE205365 which has been made publicly available.
All datasets used and/or analyzed during the study have been included in the attached Source data file.

Human research participants

Policy information about studies involving human research participants and Sex and Gender in Research.

Reporting on sex and gender	n/a
Population characteristics	n/a
Recruitment	n/a
Ethics oversight	n/a

Note that full information on the approval of the study protocol must also be provided in the manuscript.

Field-specific reporting

Please select the one below that is the best fit for your research. If you are not sure, read the appropriate sections before making your selection.

[x] Life sciences [ ] Behavioural & social sciences [ ] Ecological, evolutionary & environmental sciences

For a reference copy of the document with all sections, see nature.com/documents/nr-reporting-summary-flat.pdf

Life sciences study design

All studies must disclose on these points even when the disclosure is negative.

Sample size	No power calculations were performed for in vitro experiments. We used standard in vitro sample size of n=3 biological for BMDC derived from mice. Biological replicates of at least 3 were used for BMDC experiments. For metabolic assay studies each biological replicate had at least 3 technical replicates. Each figure contains full details regarding sample numbers for the related figure.
Data exclusions	No data were excluded from analyses.
Replication	Experiments were performed a minimum of 3 times independently. All attempts at replication were successful. Each figure legend contains details of experimental replicates for the related figure.
Randomization	Both male and female littermate mice of C57Bl/6J background were used at 6-10 weeks of age for WT BMDM generation. Our study did not involve any experiments where randomization would increase the reliability of the results.
Blinding	Blinding was not required in this study. All data were recorded and analysed under the same experimental conditions to exclude any bias.

Reporting for specific materials, systems and methods

We require information from authors about some types of materials, experimental systems and methods used in many studies. Here, indicate whether each material, system or method listed is relevant to your study. If you are not sure if a list item applies to your research, read the appropriate section before selecting a response.

Materials & experimental systems

n/a	Involved in the study
	☒ Antibodies
	☒ Eukaryotic cell lines
☒	Palaeontology and archaeology
	☒ Animals and other organisms
☒	Clinical data
☒	Dual use research of concern

Methods

n/a	Involved in the study
☒	ChIP-seq
	☒ Flow cytometry
☒	MRI-based neuroimaging

Antibodies

Antibodies used

1. Rabbit monoclonal anti phospho- p38 Thr180/Tyr182 1/1000 (Clone D3F9 - Cat: 4511; CST). 1:1000
2. Rabbit polyclonal anti p38 MAPK (p38 p38α, -β or -γ MAPK) (Cat: 9212, CST). 1:1000
3. Rabbit monoclonal anti phospho- NF-κB p65, Ser536 (Clone 93H1 - Cat: 3033, CST). 1:1000
4. Rabbit monoclonal anti NF-κB p65 Glu498 (Clone D14E12 - Cat: 8242, CST). 1:1000
5. Mouse monoclonal anti- κBα Amino-terminal Antigen (Clone L35A5 - Cat: 4814, CST). 1:1000
6. Rabbit monoclonal anti phospho- IκBα Ser32/36(Clone 5A5 - Cat: 9246, CST). 1:1000
7. Rabbit polyclonal anti SAPK/JNK (Cat: 9252, CST). 1:1000
8. Rabbit polyclonal anti phospho-JNK Thr183/Tyr185 (Cat: 9251, CST). 1:1000
9. Mouse monoclonal β-actin N-terminal (Clone AC-74 - Cat: A2228, Sigma-Aldrich). 1:3000
10. Rabbit monoclonal anti-HIF-1α Leu478 (Clone D1S7W - Cat: 36169, CST), 1:1000
11. Mouse Monoclonal anti-LAMP-1 (Clone H4A3 - Cat: sc-20011, SantaCruz) 1:50
12. Mouse IgG2 PE conjugated anti-pentahis Tag (Clone J095G46 - Cat: 362603, BioLegend,) 1:25
13. Mouse IgG2 PE Isotype Ctrl (Clone MOPC-173 - Cat: 400244, BioLegend,) 1:25
14. Mouse monoclonal anti LRP1 (Clone 8G1 – Cat: ab20384, Abcam) 1:1000
15. Mouse monoclonal anti-LRP1 (Clone LRP1-11 – Cat: L2420, Sigma) 1:100
16. Rat IgG2 anti-CD11b PE (Clone M1/70 – Cat: 101207, Biolegend,) 1:20
17. Rat IgG2 anti CD206 brilliant violet 421 (Clone C068C2 - Cat: 141717, Biolegend) 1:20
18. Rat IgG2 Isotype Ctrl PE (Clone RTK4530 – Cat: 400636, Biolegand) 1:20
19. Rat IgG2 Isotype Ctrl brilliant Violet 421 (Biolegend, 400535) 1:20
20. Rat IgG2 anti-CD38 APC, (Clone 90 - Cat: 102712, Biolegend) 1:20
21. Rat IgG2 Isotype Ctrl APC (Clone RTK2758 – Cat: 400511, Biolegend) 1:20
22. Polyclonal Fc Receptor Binding Inhibitor Antibody (Cat: 14-9161-73, eBioscience) 1:20
23. Polyclonal anti-rabbit IgG AlexaFluor 488 (Cat: A-11008, ThermoFisher Scientific) 1:1000
24. Polyclonal anti-mouse IgG AlexaFluor 594 (Cat: A-11032, ThermoFisher Scientific) 1:300
25. Polyclonal anti-rabbit IgG HRP-conjugated Antibody (Cat: HAF008, R&D Systems) 1:1000
26. Polyclonal anti-mouse IgG HRP-conjugated Antibody (Cat: HAF007, R&D Systems) 1:1000
27. Polyclonal peroxidase AffiniPure rabbit anti-Goat IgG (H+L) HRP-conjugate Antibody (Cat: 305-035-003, Jackson Immunoresearch) 1:5000
28. Polyclonal rabbit anti-human VWF Antibody (Cat: GA52761-2, Agilent DAKO) 1:1000
29. Monoclonal mouse anti-EEA1 Antibody (Clone 14/EEA1-Cat: 610457, BD Biosciences) 1:100
30. Rat IgG2b Fc-block anti-mouse CD16/CD32 mAb (Clone FRC-4G8 - Cat: 10291623; Invitrogen) 1:100
31. Mouse IgG1 anti- mouse F4/80-FITC mAb (Clone QA17A29 - Cat 157313; Biolegend) 1:20
32. Mouse IgG2a anti- mouse Siglec F-APC mAb (Clone FRC-4G8 - Cat 14-1702-82; Invitrogen 1:20
33. Rat IgG2b anti-mouse/human CD11b-APC-Cy7 mAb (Clone M1/70 - Cat 101225; (Biolegend) 1:20
34. Rat IgG2a anti-mouse Ly6G-BV650 mAb (Clone 1A8 - Cat 127625; Biolegend) 1:20
35. Mouse IgG1 anti-mouse CD45-BV711 mAb (Clone 1A8 - Cat 157602; Biolegend) 1:20
36. Mouse IgG2a anti-mouse MHC-II-PE mAb (Clone AF6-120.1 - Cat 116419; Biolegend) 1:20
37. Life/Dead Aqua (Cat L34957; Thermo Scientific) 1:1000

Validation

Antibodies were selected based on prior publications and from reputable sources. Antibodies were validated already on manufacturers' websites. Pilot experiments were performed to confirm validation under our experimental conditions.

Eukaryotic cell lines

Policy information about cell lines and Sex and Gender in Research

Cell line source(s)

THP1 and HEK293T cell lines were obtained from ATCC.

Authentication

THP1 cell phenotype before and after macrophage differentiation and HEK293T cells were assessed using flow cytometry as detailed in the manuscript.

Mycoplasma contamination	All cell lines tested negative for mycoplasma
Commonly misidentified lines (See ICLAC register)	None

Animals and other research organisms

Policy information about studies involving animals; ARRIVE guidelines recommended for reporting animal research, and Sex and Gender in Research

Laboratory animals	Adult 8 to 12 weeks old C57BL/6J strain mice were used in all experiments. Animals were housed in a specific pathogen-free facility in individually ventilated and filtered cages under positive pressure.
Wild animals	No wild animals were used in the study.
Reporting on sex	Both male and female littermate mice of C57Bl/6J background were used at 6-10 weeks of age for wild type BMDM generation. Preliminary experiments did not show any differences according to sex.
Field-collected samples	No field collected samples were used in the study.
Ethics oversight	All animal experiments were approved by the Animal Research Ethics Committee, Trinity College Dublin, and under license from the Ireland Health Products Regulatory Authority (AE19127/P060). All procedures conformed to the Directive 2010/63 EU of the European Parliament.

Note that full information on the approval of the study protocol must also be provided in the manuscript.

Flow Cytometry

Plots

Confirm that:

- [x] The axis labels state the marker and fluorochrome used (e.g. CD4-FITC).
- [x] The axis scales are clearly visible. Include numbers along axes only for bottom left plot of group (a 'group' is an analysis of identical markers).
- [x] All plots are contour plots with outliers or pseudocolor plots.
- [x] A numerical value for number of cells or percentage (with statistics) is provided.

Methodology

Sample preparation

VWF Binding
Differentiated THP1 cells were detached from petri dishes by continuous ice cold PBS pipetting and subsequently divided between treatments (5x105 cells/flow tube). Alternatively, non PMA treated suspension monocytic THP1 cells were used. VWF variants of interest were incubated with THP1 macrophages in binding buffer (Hanks balanced salt solution supplemented with HEPES (10mM), MnCl2 (1mM) and CaCl2 (1mM) ± ristocetin (Helena, UK 1.5mg/ml) where indicated) for 30 minutes at room temperature. Cells were washed once in 2ml of ice cold binding buffer. FC-receptors were blocked for 10 minutes on ice. Where applicable, THP1 cells were washed with 2ml of binding buffer between and after 30 minute incubations (on ice) with primary and secondary antibodies. Following staining, cells were fixed in binding buffer containing 1% PFA for 10 minutes on ice.

Human primary macrophages were differentiated from buffy coat isolated CD14High monocytes on 48 well low adherent plates (Starstedt, Germany) and detached by gentle scraping with the barrel of a 1ml syringe. Cells were pooled and equally divided between treatment flow tubes (2x105-1x106). VWF was incubated for 30min at room temperature and cells were washed with room temperature RPMI Phenol red free, supplemented with CaCl2 (1mM). FC-receptors were blocked for 10 minutes, and without washing cells were incubated with anti-vwf for 30mim. Cells were washed RPMI Phenol red free, supplemented with CaCl2 (1mM) before and after a 30min incubation with Anti-Rabbit Alexa Fluor 488. Following staining, cells were fixed in binding 1% PFA for 10 minutes on ice.

M1 and M2 Populations
BMDM were washed with RPMI++ media, lifted by lite scraping with the barrel of a 1ml syringe. Fc receptors were block for 10min on ice, and without washing cells were stained(and IgG control) for 30 minutes on ice. Cells were washed in RPMI++ then fixed in 1% PFA for 10 minutes on ice and washed again.

Murine Lavage Fluid
Lavage fluid was harvested and cells were washed in 5% FBS in RPMI phenol red free and subsequently Fc blocked was added for 10 min before staining. Without washing cells were stained (and IgG controls) for 30min in 5% FBS in RPMI phenol red free on ice. Cells were washed in 5% FBS in RPMI phenon red free, then fixed in 1% PFA for 10 minutes. Cells were washed once before running.

Reactive oxygen sepsis

	BMDMs were washed in RPMI++ supplemented with CaCl2 (1mM) and incubated with CellRox Deep Red (5µM, ThermoFisher Scientific, USA) and LiveDead stain (1/1000, TheremoFisher Scientific, USA) for 30 minutes at 37^0C. Prior to staining a control dead cell population was determined using 70% ethanol.
Instrument	BD Facs CANTO II and CyAn Beckman Coulter
Software	Systems were run using BD FACS Diva and Summit respectively. Data analised using FlowJo.
Cell population abundance	Human BPBMC were isolated by histopaque gradient 1077, (Sigma-Aldrich) and monocytes subsequently isolated using CD14 magnetic positive selection MicroBeads ((Miltenyi Biotec, UK). Monocytic population was confirmed by detecting CD14 and CD16 expression by flow cytometry and was >95%,. Monocytes were differentiated into macrophage, which was determined by high CD11b expression. Minimum of 10,000 events were captured within the final gate of interest.<br><br>BMDMs were purified from bone marrow by M-CSF selection, and confirmed by >90% CD11b expression. Minimum of 10,000 events were captured within the final gate of interest.<br><br>Total Lavage cells obtained per mice were and on average 2x10^6 cells. Average of 4 mice lavage control gate of interest extended time, cells percentage: NK Cells 12.6, DC 4.9, Nuetro 0.278, Mac 29.95 (MHCII++ 17.89, MHCII- 82.1) and T cells 6.54 Average of 4 mice lavage VWF gate of interest extended time, cell percentage: NK Cells 10.91, DC 2.99, Nuetro 4.89, Mac 18.82 (MHCII++ 35.77, MHCII- 64.25) and T cells 6.78.<br>Average of 4 mice lavage control gate of interest short time, cells percentage: NK Cells 0.33, DC 2.687, Nuetro 0.52, Mac 16.75 and T cells 14.25 .Average of 4 mice lavage VWF gate of interest short time, cell percentage: NK Cells 1.012, DC 1.572 Nuetro 23.492, Mac 3.55 and T cells 8.29.
Gating strategy	M1 and M2 populations<br>Live cells were determined by forward scatter side scatter, and single cells isolated by pulse with. Background florescence was determined by IgG controls. Macrophages were subsequently identified as CD11b positive cells.<br>CD11b positive were then gated for CD206 and CD38 expression.<br><br>VWF Binding<br>Live cells were determined by forward scatter side scatter and single cells isolated by pulse with. Normal PE-IgG control was used to determine nonspecific binding. Baseline fluorescence was established using 0µg/ml VWF incubated with PE conjugated anti penta-his tag.<br><br>Reactive oxygen sepsis(ROS)<br>Cells were gated on forward scatter, side scatter. Single cells were isolated using CANTOs forward scatter area Vs forward scatter height. Subsequently, the dead cell population was excluded based on FITC positive cells, and FITC negative cells were selected. ROS was determined by FITC negative, red positive(Cells rox) fluorescent signal.<br><br>Please see attached for gating of in-vivo panel. Cells were first gated on side and forward scatter, and single cells were determined by pulse with. Compensation was determined by single stained cells, and non specific binding determined by florophore conjugated IgG control.

☒ Tick this box to confirm that a figure exemplifying the gating strategy is provided in the Supplementary Information.
